# Wearable flexible body matched electromagnetic sensors for personalized non-invasive glucose monitoring

**DOI:** 10.1038/s41598-022-19251-z

**Published:** 2022-09-01

**Authors:** Jessica Hanna, Youssef Tawk, Sami Azar, Ali H. Ramadan, Batoul Dia, Elias Shamieh, Sumaya Zoghbi, Rouwaida Kanj, Joseph Costantine, Assaad A. Eid

**Affiliations:** 1 Biomedical Engineering Program, American University of Beirut, Riad El Solh Street, Beirut, 1107 2020 Lebanon; 2 Department of Electrical and Computer Engineering, Maroun Semaan Faculty of Engineering and Architecture, American University of Beirut, Riad El Solh, Beirut, 1107 2020 Lebanon; 3 Department of Anatomy, Cell Biology, and Physiological Sciences, Faculty of Medicine, American University of Beirut, Riad El Solh, Beirut, 1107 2020 Lebanon; 4 The AUB Diabetes Program, Faculty of Medicine and Medical Center, American University of Beirut, Riad El Solh Street, Beirut, 1107 Lebanon

**Keywords:** Diabetes, Biomedical engineering, Electrical and electronic engineering

## Abstract

This work introduces novel body-matched, vasculature-inspired, quasi-antenna-arrays that act as electromagnetic sensors to instantaneously, continuously, and wirelessly sense glucose variations in the bloodstream. The proposed sensors are personalized, leverage electromagnetic waves, and are coupled with a custom machine-learning-based signal-processing module. These sensors are flexible, and embedded in wearable garments such as socks, which provide conformity to curved skin surfaces and movement resilience. The entire wearable system is calibrated against temperature, humidity, and movement resulting in high accuracy in glucose variations tracking. In-Vivo experiments on diabetic rats and pigs exhibit a 100% diagnostic accuracy over a wide range of glucose variations. Human trials on patients with diabetes and healthy individuals reveal a clinical accuracy of continuous glucose monitoring of 99.01% in twenty-eight subjects who underwent Oral Glucose Tolerance Tests. Hence, our approach ensures the continuous tracking of glucose variations from hypo-to-hyper glycemic levels with great fidelity.

## Introduction

The future of continuous glucose monitoring is confidently heading towards non-invasive wearable solutions that are needle-free and pain-free, as also supported by the recent report of the world economic forum on the top ten emerging technologies^1^. It is now clear that continuous monitoring of the glycemic profiles of patients with diabetes, delays the progression of the disease^2,3^ since it provides a significant amount of information that allows healthcare professionals to plan personalized diabetes treatment regimens based on the patient’s individual glycemic profile. All the developmental efforts must focus on providing the patients with continuous glucose monitoring (CGM) solutions that are not dependent on finger pricking for blood extraction but rather on a painless wireless method to detect glucose variations^4^. In fact, studies show that conventional blood glucose monitoring techniques that are painful such as finger pricking, result in non-compliance with regular glucose monitoring within the diabetic patients community^2,4^. This is a consequence that can be dire to many and may result in missing critical hypo and hyperglycemic events.

The work from earlier efforts resulted in minimally invasive sensors, which depend on short-term microneedles inserted inside the skin. These sensors rely on amperometric enzyme electrodes that last only up to 14 days. They interpret the acquired data and translate them into dynamic plots providing information about the rate and the direction of glucose change during the day, month, and year. Despite its good accuracy, this approach can still be considered uncomfortable and needs additional effort to reduce any undesired discomfort from irritation or other predicaments at the insertion location^5^. It can increase the patient’s resistance especially for elderly and kids, and most importantly, it still suffers from high socio-economic burdens^6,7^.

Hence, the ability to cater for the future of glucose monitoring by presenting a non-invasive, easy to use, and relatively low-cost technology will be game changing and life altering for many patients with diabetes. Beyond that, positioning the patient’s comfort and preference as a priority in the design process is needed to enhance the quality of life for millions around the world and to allow clinicians to optimize diabetes therapeutic treatments. However, it remains extremely challenging to achieve CGM noninvasively. During the past decade, researchers studied several noninvasive glucose monitoring techniques as detailed in supplementary Note 1. These techniques include infrared spectroscopy^8^, ultrasound^9,10^, fluorescence^11^, optical coherence tomography^12^ and reverse iontophoresis^13^. Each of these solutions offer several advantages but all suffer from several technical challenges. These technology-dependent limitations include lack of specificity, skin irritation, serious time lag when measuring glucose in the interstitial fluids, poor correlation between the measured glucose levels and the blood glucose levels when monitoring the glucose in tears or saliva and lack of stability due to interference from confounding factors^13–17^.

Dielectric spectroscopy is an approach where electromagnetic (EM) systems are recently studied for continuous, noninvasive glucose monitoring through skin and underlying tissues^18–23^. Since every biological tissue has a unique dielectric signature, it can be characterized by frequency dependent parameters such as permittivity and conductivity. By observing the unique dielectric spectrum and their changes, the concentration of glucose can be detected^24–26^.

**Figure 1:**
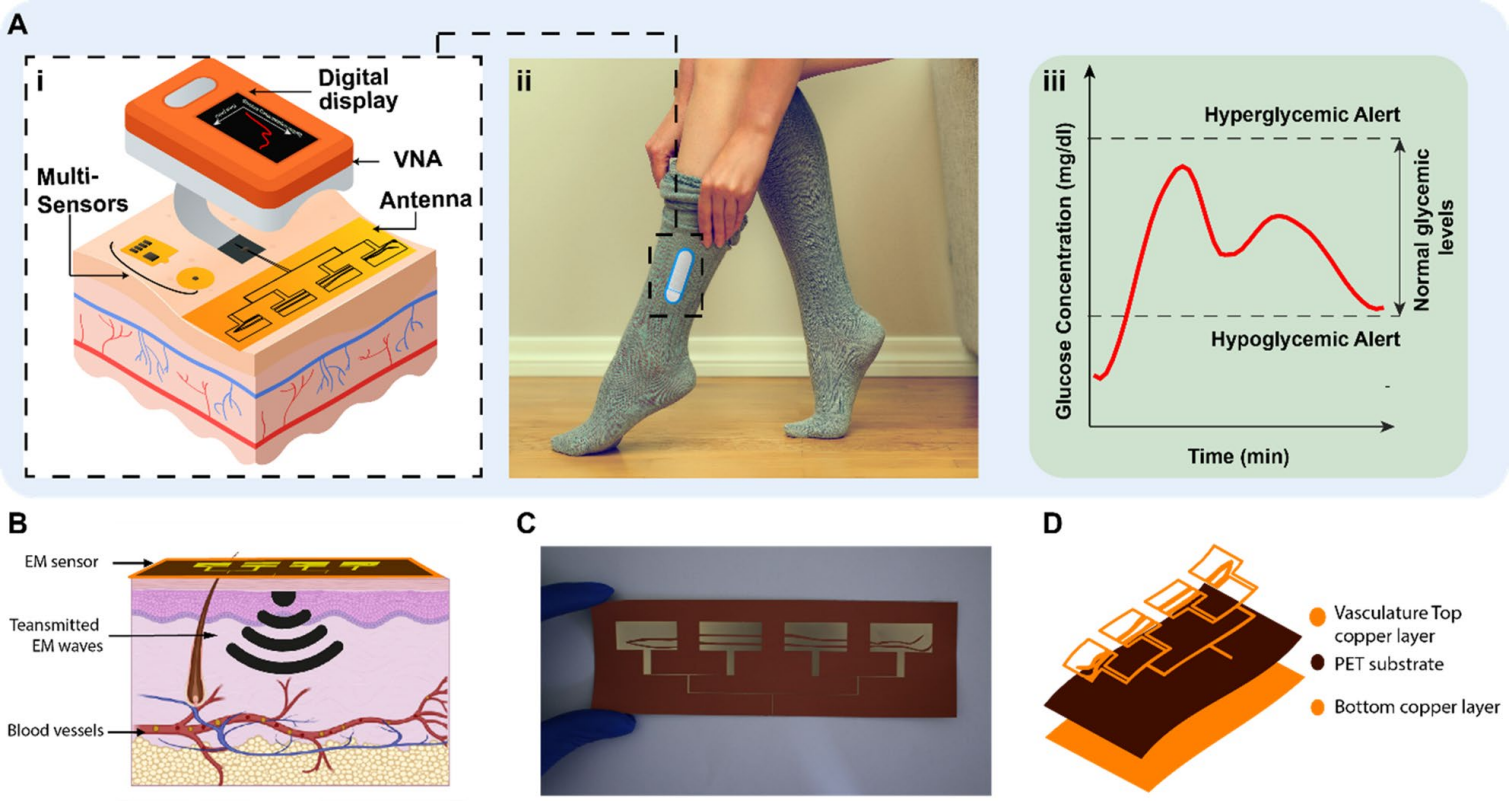
Principle of a multi-sensing, multi-location approach. (**A**), i, the system is composed of two sensors targeting two on-body locations. Additional environmental and physiological sensors are added to the system to calibrate the temperature, humidity, sweat, and motion effects. ii, all these sensors are worn within the sock apparatus. **iii**, the system provides the glucose levels in mg/dl using a machine learning algorithm. (**B**) Targeted blood vessels to provide information about the blood dielectric properties. The reflected EM waves are monitored and correlated to the blood glucose variation. (**C**) Top sensing layer of the flexible leg sensor prototype. (**D**) The layer-by-layer layout of the integrated leg sensors where the bottom layer is composed of copper, the middle layer is a PET substrate and the top layer is a copper layer.

However, most of the proposed EM based devices rely solely on EM sensors. Nevertheless, in daily-life situations, dielectric spectroscopy could be affected by a variety of environmental and physiological factors^17^. These perturbing factors include ambient temperature and humidity, skin temperature, skin conductance that is mainly affected by sweat, and motion which could affect the sensor-skin contact. Hence, taking into consideration all these different perturbing factors significantly enhances the accuracy and efficiency of EM based noninvasive glucose monitoring systems especially when tested on patients with diabetes. To this end, we developed a fully non-invasive continuous glucose monitoring system that leverages electromagnetic and radio frequency (RF) technology in sensors with topologies that mimic the vasculature anatomy of a human leg. The sensors are proposed to be integrated in socks that can be worn by patients with diabetes. Furthermore, the response from these wearable devices is correlated with multiple environmental sensors to calibrate against perturbing factors such as temperature, mobility, and humidity as shown in Fig. 1A. All these sensors are worn within the sock apparatus and add to the accuracy of the proposed EM sensor’s response. Moreover, the sock integrated sensing system was tested in conjunction with a sensor integrated in a glove introduced in^27^, resulting in a robust system of non-invasive glucose sensing that extends its capability beyond all available non-invasive sensing techniques. The sensing system exhibited a picomolar sensitivity in different experimental setups. The whole system was tested on serum, animal models of diabetes and in a clinical setting on control individuals and patients with diabetes. Such a system has displayed 99.01% accuracy with an ability to track glucose variations from hypo- to hyperglycemic ranges.

## Results

### Electromagnetic simulation of the EM-sensors and fabrication process

The EM-based multi-sensing system comprises of three main modules as shown in Fig. 1:

1. A Glucose sensing module, which is composed of EM-based sensors to monitor the glucose with picomolar accuracy;
2. An environmental and physiological sensing module, which calibrates out the potential perturbing factors in real time;
3. A signal processing module to process the output of the multiple integrated sensors along with a regression model to convert the readings into absolute glucose levels.

The EM sensors are designed to target different body locations simultaneously. Electromagnetic waves are transmitted to the body where the reflected and the transmitted waves are impacted by the underlying tissues. More specifically, changes in the scattering parameters (magnitude and phase) of the reflected and\or transmitted waves are associated with the glucose fluctuations in the medium under test as detailed in supplementary Notes 2–4. By monitoring and analyzing these changes, variations in blood glucose levels can be detected. The glucose sensing module is composed of a multi-band flexible microstrip quasi-antenna-array targeting the leg’s vessels, proposed to be integrated in a sock, which can be used alone or in conjunction with a multi-band slot antenna that targets the hand vessels. We demonstrate that such diverse incorporation of the sensing components in multiple locations across the body enables a higher accuracy in tracking blood glucose levels. To test and compensate for the possible influence of perturbing factors such as temperature, humidity, skin sweat as well as body movements, on the glucose sensors’ readings, we designed a sensing module comprising: a skin temperature sensor^28^, a skin conductance response (SCR) sensor^29^, an environmental temperature and humidity sensor^30^ and a motion sensor^31^. Each sensor provides non-invasive and continuous monitoring of these different perturbing factors resulting in a holistic glucose sensing system.

The data collected includes the reflection coefficients from the glucose sensors along with readings from the environmental/physiological sensors. The data is processed and integrated into a multivariate Gaussian Processes regression model^27^ to predict the absolute glucose levels as shown in Fig. 1A. More details about the data processing are presented in supplementary Note 8–9.

### Operational characteristics of EM-sensors and response to glucose

**Figure 2:**
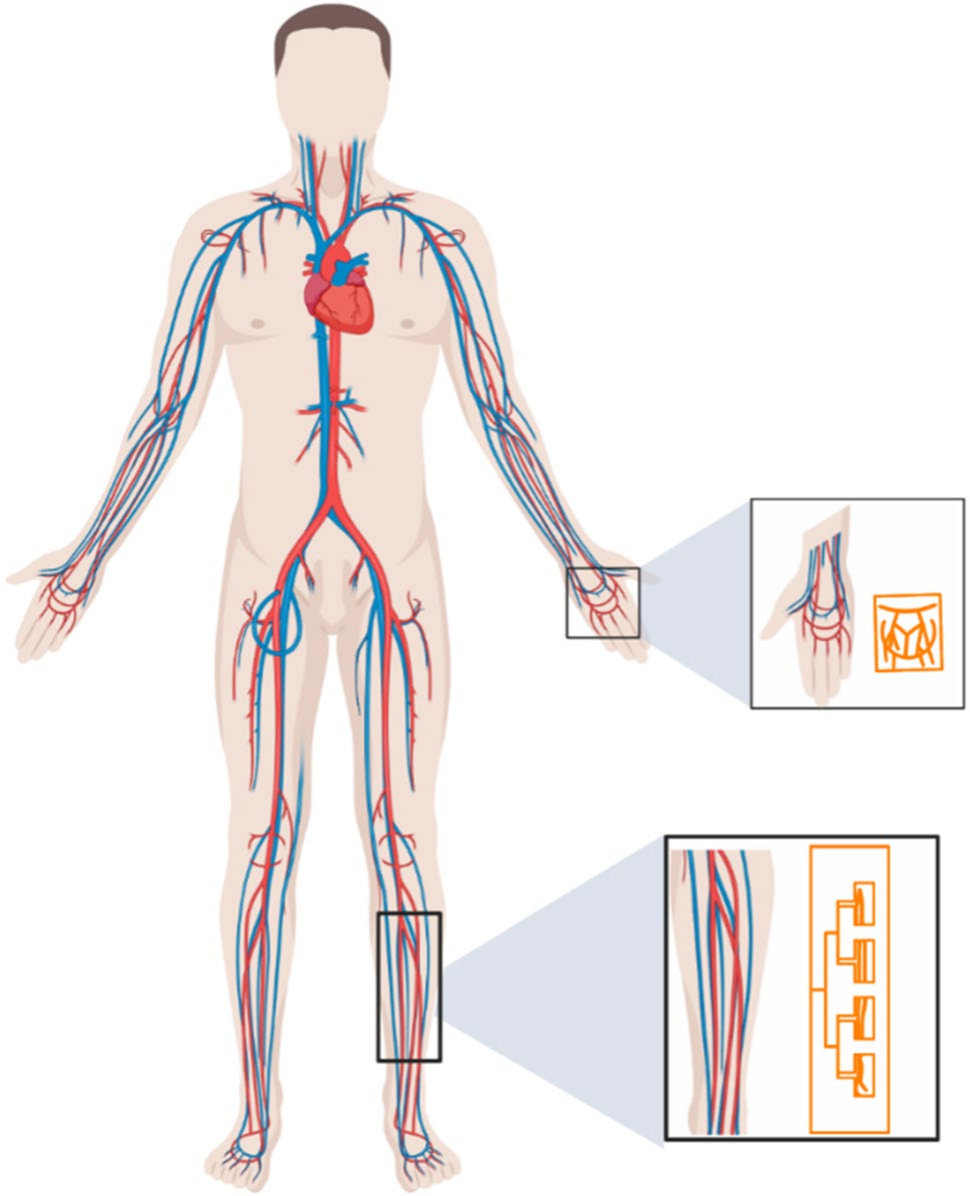
The sensors are biologically inspired by the vasculature anatomic of the targeted blood vessels. The EM hand sensor is a multi-band slot antenna. The top sensing layer is composed of slots following the main arteries and veins of the hand. The EM leg sensor is a microstrip patch quasi-antenna-array. The radiating slots are inspired by the shape of the arteries and veins in a human lower leg.

The EM sensors are designed with topologies that mimic the vessels corresponding to their respective body locations. For example, the EM sensor integrated in a sock is designed with a topology that mimics the leg vessels, while adopting a quasi-antenna-array form factor (Fig. 2). The proposed sensors operate at multiple frequencies ranging between 0.5 and 4 GHz. The EM waves at this frequency range can effectively penetrate human tissues, which allows, due to the optimized sensor topologies, an enhanced exposure of the blood stream to the EM signals. The properties of the reflected waves, in terms of scattering parameters or S-parameters, indicate the changes in terms of dielectric properties of the material under test and hence these parameters are utilized to monitor the glucose levels in the blood. The sensors are on-body matched, and they are designed when loaded with human models. In other words, the human body is part of the sensor design, and the sensor is impedance matched to its corresponding body location. This characteristic reduces the reflection of the EM waves at the skin-air boundary and improves the penetration of the waves, to enable an easier reach of the targeted arteries and veins.

Each EM sensor is composed of three layers; the top sensing layer is composed of the vessels-like slots, which allow the concentration of the EM waves onto the targeted veins and arteries along with the feeding structure. For the EM leg sensor, the radiating slots are inspired by the shape of the peroneal venae comites and posterior tibial venae comites shown Fig. 2. The sensor is used to transmit electromagnetic waves into human tissues in areas near the main vanea comites veins. These multiple slots also result in a multiband frequency behavior, which enables monitoring the glucose variations at multiple frequencies simultaneously. The middle layer comprised the flexible Polyethylene Terephthalate (PET) substrate. With this ultrathin substrate, the wearable EM sensors can ensure conformal intimate contact with the curvilinear skin surface. The bottom layer comprises a copper ground plan. The sock containing the designed antenna ensures comfort and discretion for patients with diabetes.

### In-vitro measurements of glucose with leg EM sensor

**Figure 3:**
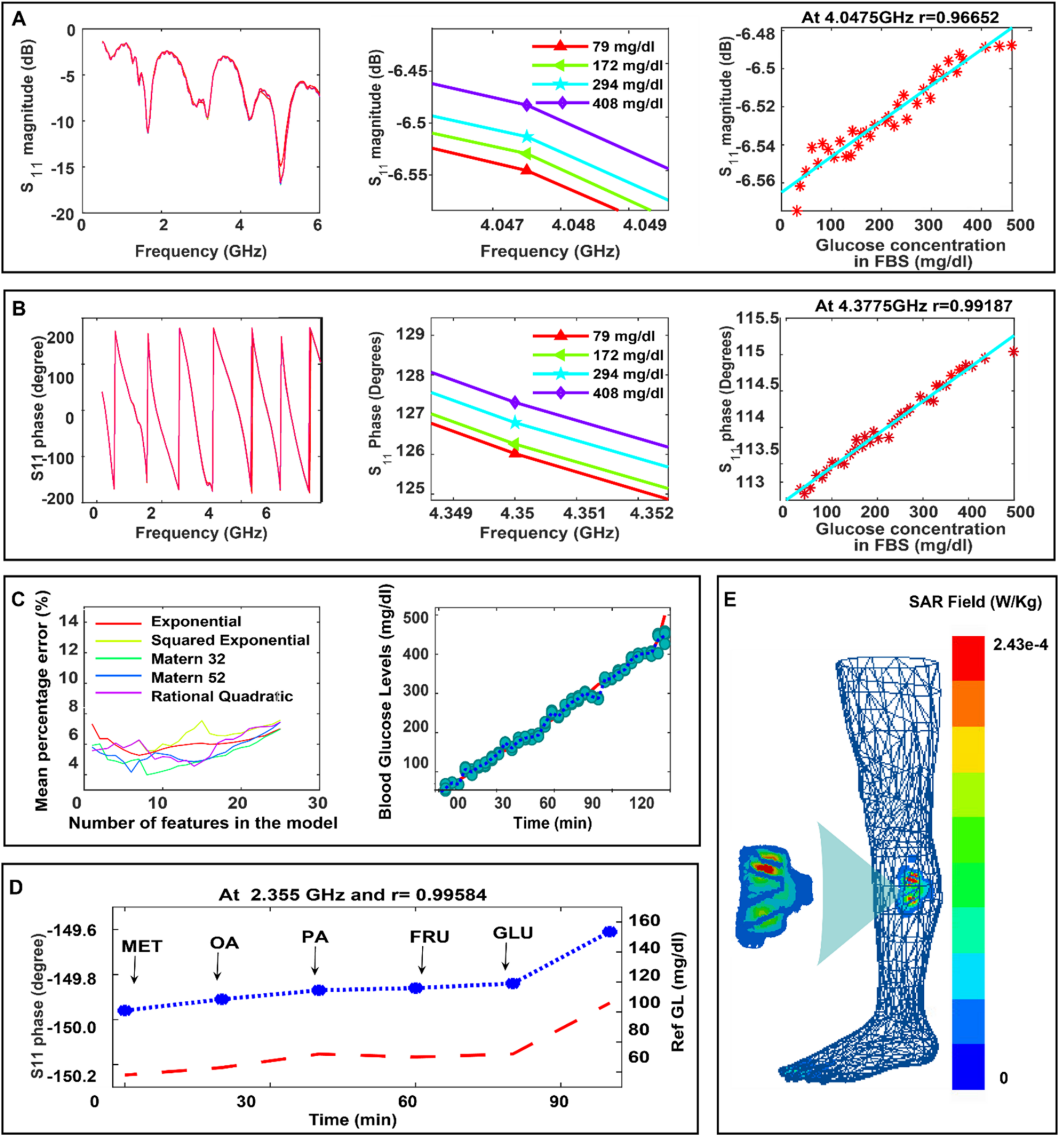
EM Leg sensor's response to glucose variation during in-vitro experiments. (**A**) Left: The sensor antenna’s reflection coeffcient’s magnitude response (S11 VS frequency) for different glucose levels. Middle: A zoomed-in view of the sensor antenna’s S11 magnitude response around a frequency of 4.04 GHz. Right: The S11 Magnitude versus the reference glucose levels. The cyan line is the S11 fitted curve showing the trend of the sensor antenna’s response when the glucose levels increase. (**B**) Left: The sensor antenna’s reflection coefficient’s phase response (S11 VS frequency) for different glucose levels. Middle: A zoomed-in view of the antenna’s S11 phase response around 4.3 GHz. Right: The S11 phase versus the reference glucose levels. (**C**) The prediction of the glucose levels. Left: Wrapper feature/kernel selection results. Right: The non-invasively estimated GL versus the reference GL. (**D**) Selective response of the flexible antenna to glucose (Glu). (**E**) Skin SAR Plot with full leg model in function of frequency for the EM leg sensor design. The peak SAR averaged over 1 g of tissue is 2e−4 W/kg obtained at 3.3 GHz.

To characterize the sensitivity of the proposed EM leg sensor (quasi-antenna-array), we tested it first with fetal bovine serum^32^ (FBS) spiked with known concentrations of glucose (Supplementary Fig. S1). We introduced increased doses of glucose to the solution and used the proposed EM leg sensor to monitor glucose changes for approximately 8 h. Over the course of the experiment, we started with a glucose level of 40 mg/dl (2.22 mmol/l) that reached 500 mg/dl (27.75 mmol/l) at the end of the experiment. The S11 raw responses of the antenna presented in Fig. 3A,B show that our proposed EM-leg sensor can detect picomolar changes in glucose levels. The raw data collected from our proposed noninvasive EM sensor closely follows the reference glucose levels obtained by a commercially available finger-prick glucometer^33^. As a result, the proposed EM leg sensor can achieve a correlation of 0.96 and 0.99 between the reference glucose levels, S11 magnitude and S11 phase respectively as shown in Fig. 3A,B. More details about the in-vitro experiment are presented in supplementary Note 10.

Beyond regular glucose changes, this experiment highlights the ability of the proposed EM leg sensor to detect glucose levels that vary from hypoglycemic to hyperglycemic ranges. We were able to identify multiple frequencies at which the physical parameters of the proposed sensor exhibited a heightened sensitivity towards the glucose variations. This has essential implications for patients, who are naturally diverse, and have different underlying tissue compositions and hence respond differently to the EM-waves application. Thus, it is important to provide personalized monitoring and find the glucose- sensitive frequency that suits each patient. Through this approach, the patient has been put at the center of the design.

In addition, we developed a custom signal-processing module to process and predict the glucose levels based on the data collected from the different sensors (See Data collection and feature selection in the methods). Figure 3C shows the prediction results for the EM leg sensor. Figure 3C left shows the mean percentage error in function of the number of features used by the model for the different kernel functions using the wrapper method as a feature selection technique. More details about data collection and feature selection are presented in supplementary notes 8–9. It’s clear that the mean percentage error, or cross validation error, decreases when more features are added to the model until it reaches a minimum value and then it starts to increase again. The wrapper technique resulted in a mean percentage error, which dropped, from 9% to around 4% for the best number of features and kernel function (9 features, matern32 as kernel function). Figure 3C right presents estimated glucose levels versus the reference glucose levels using Gaussian process regression models for the EM leg sensor. The squared exponential kernel provided the lowest mean percentage error using 15 features, achieving a mean absolute relative difference (MARD)^34^ of only 8.25%.

### Response to common interferants

To evaluate the effect of common interferants observed during diabetes, including the use of Metformin (MET), Oleic acid (OA), Paracetamol (PA) by the patient as well as other glucose like molecules such as Fructose (FRU), on the proposed sensor’s response, glucose (GLU) and these interferants were added to an FBS solution in concentrations much higher than their physiological ranges. More details about the experimental setup are presented in supplementary Note 5. We added successively 50 mg/dl (2.775 mmol/l) of MET, OA, PA, FRU and GLU to the same FBS solution. The S11 parameters showed minimal to no shift when the interferants were added (Fig. 3D). In contrast, a significant shift of S11 parameters was produced when the same amount of glucose was added to the solution, resulting in a correlation with the glucose levels of R > 0.9 for the proposed EM leg sensor.

### Response to movement

In practical scenarios, the wearable nature of the design limits the room for misalignment between the slots of the EM sensor and the targeted veins and arteries. Moreover, the surface current, shown in Supplementary Fig. S2, is concentrated not only on the slots but rather around them. This helps accommodate for any possible slight misalignment between the targeted vessels and the slots. This was proven in a serum experiment where misalignment was introduced between the slots and a vessel-like foam. During this experiment, the foam container was slightly shifted to the right. The sensitivity of the sensor was preserved as shown in Supplementary Fig. S3. More details about the experimental setup are presented in Supplementary Note 6.

### Safety consideration

For the EM leg sensor composed of the quasi-antenna-array, SAR is simulated using Ansys Electronics Desktop^35^ when the leg model is placed above the sensor and for an input power (pin) of − 15 dBm (1mW) which is equivalent to the power delivered to the antenna by the portable VNA during experiments. We notice that over the whole radio frequency range of interest, ranging from 0.5 GHz to 4 GHz, the peak SAR value is 2.43e–4W/Kg at 3.3 GHz averaged over 1 g of tissue per the US guidelines fulfilling’s the safety guidelines defined by FCC^36^ as shown in Fig. 3E. More details are presented in Supplementary Note 7 and supplementary Fig. S4. It’s important to mention that during the clinical trials, volunteers didn’t report any discomfort and no visible effect of the EM waves on the skin is observed.

### Continuous measurement of glucose in diabetic and non-diabetic animal models (rats and pigs)

**Figure 4:**
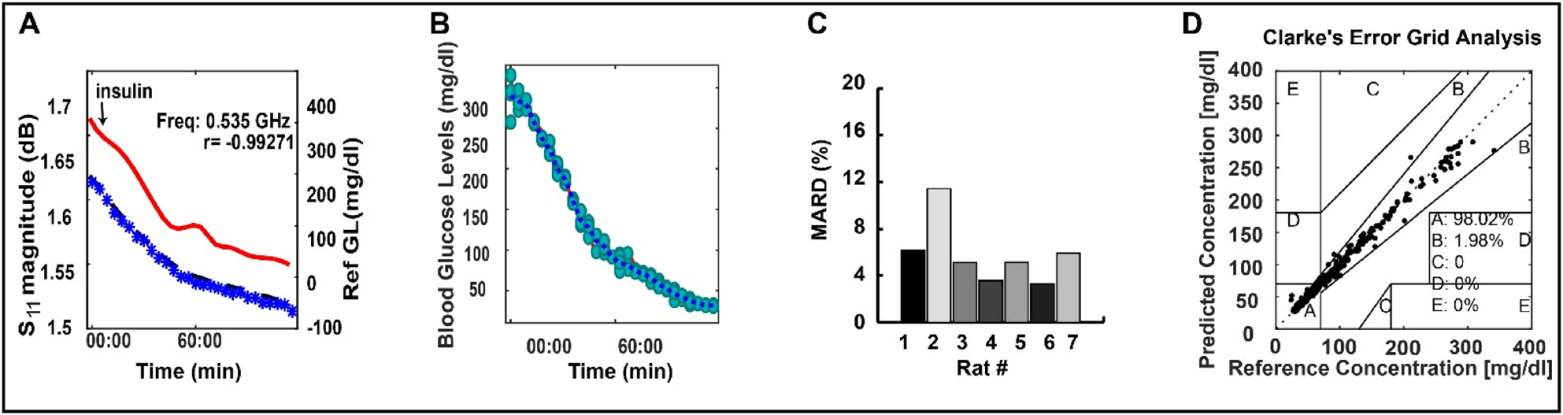
In-vivo detection of glucose levels in rats. (**A**) In-vivo experiment of one of the diabetic rat models showing the sensor’s raw S11 parameters. (**B**) Predicted glucose level. (**C**) The MARD for the experiments conducted on seven different rats. (**D**) CEG analysis for the seven experiments. Additional results are provided in the Supplementary Fig. S5.

The main purpose of the in vivo animal experiment is to test the sensitivity of the sensor to detect the hypo-and hyperglycemic events that can occur in a diabetic individual before moving to clinical trials. It’s important to mention that It is not necessary for the sensor slots to cover the underlying veins and arteries of the animal used. This is due to the fact that the surface currents are concentrated around the slots of the sensor at different frequencies, as shown in the supplementary Fig. S2. For in-vivo examination, we first tested the EM leg sensor on diabetic and non-diabetic rat models. The quasi-antenna-array was fixed on the abdomen of the anaesthetized rats. Insulin was injected into the lateral tail vein 10 min after the onset of the sensor recording. The glucose began to drop 2 min after insulin injection, and it reached a minimum of 15 mg/dl (0.8325 mmol/l) around 2 h later, as shown in Fig. 4A,B. Diabetic rat models were examined using the same procedure. Data are shown in Fig. 4 and Supplementary Fig. S5. The mean percentage error between the reference glucose levels and the predicted ones, shown in Fig. 4C, was 5.92 ± 5.3% (mean ± s.e.m., n = 7). In Fig. 4D, the Clarke’s error grid (CEG)^37^ analysis for all the experiments, which provides a measure of performance while taking into consideration the clinical relevance of the deviation between the prediction and the reference concentration, shows that the predictions are in good agreement with the reference glucose levels with 100% of the predictions falling in the clinically acceptable Zones (Zones A and B). The similarity between the results indicates that the reflected waves can penetrate the different tissues, reaching the targeted blood vessels, and providing information directly of blood glucose dielectric properties variations. This is mainly related to the appropriate radio frequency choice of the EM leg sensor, taking into consideration the targeted location.

**Figure 5:**
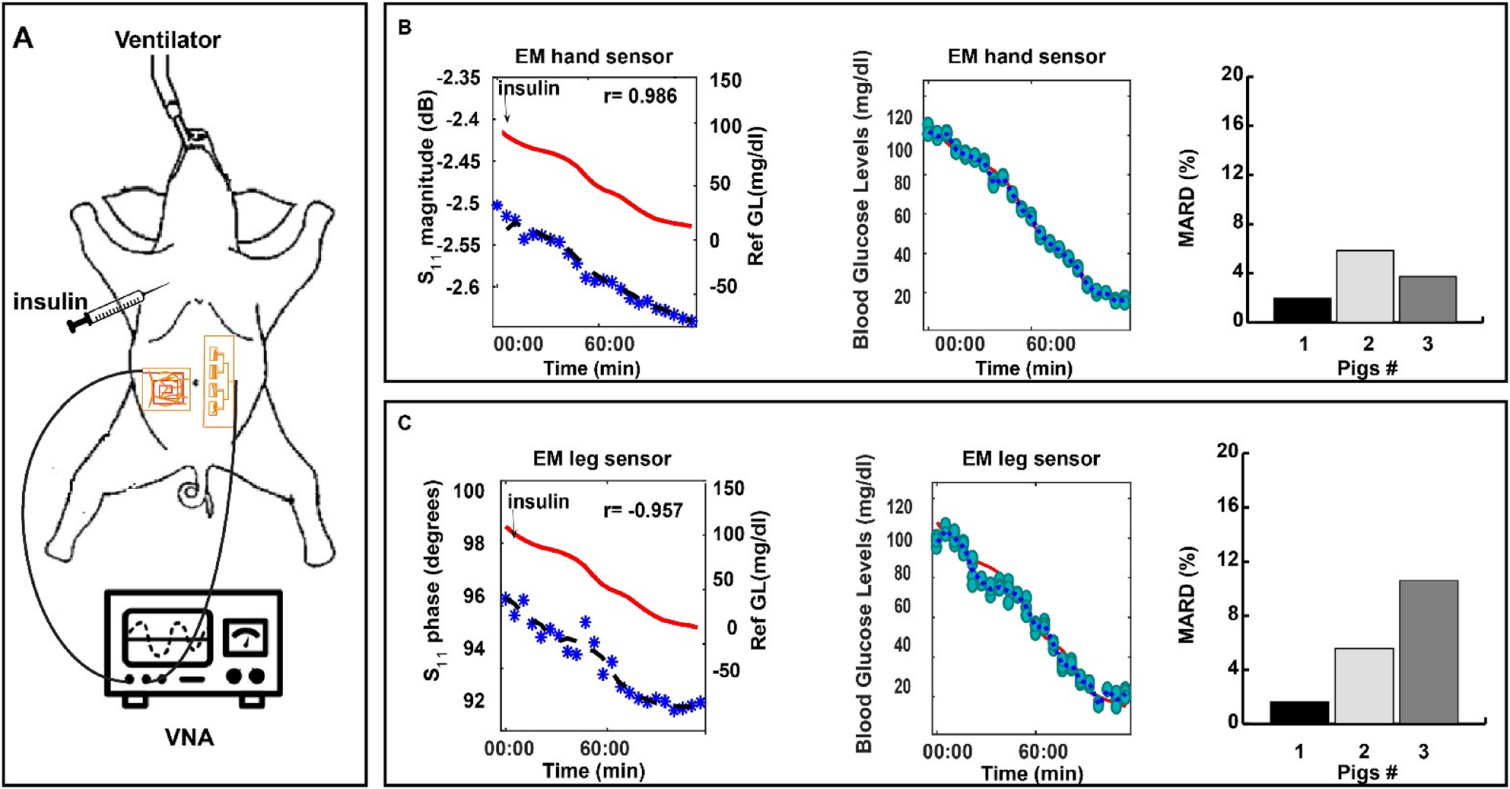
In-vivo experiments on pigs using the hand and the leg sensors. (**A**) Experimental setup. (**B**) EM hand sensor response during one experiment. Left, the raw antenna’s S11 parameters. Middle, predicted glucose levels. Right, MARD between the reference glucose levels and the predicted levels for the three conducted experiments. (**C**) EM leg sensor response during one experiment. Left, the raw antenna’s S1 parameters. Middle, predicted glucose levels. Right, MARD for the three conducted experiments.

**Figure 6:**
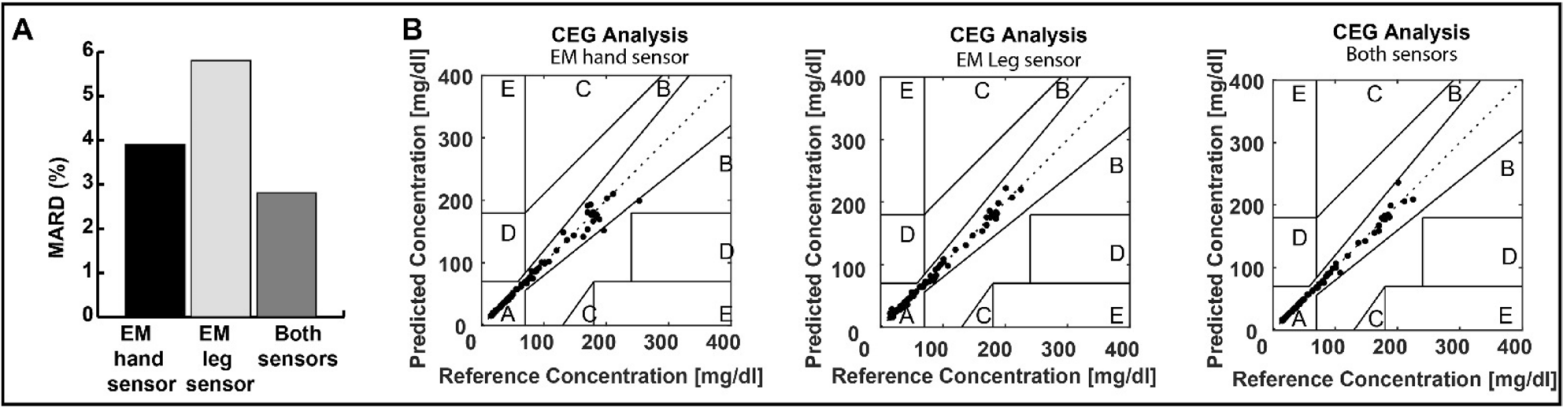
Comparison between the performance of the leg and hand EM sensors for the conducted experiments on three pigs.

Further, in-vivo experiments were performed on pigs (n = 3) as detailed in Figs. 5 and 6 and supplementary Fig. S6. The leg sensor and the hand sensor^27^ were placed in close proximity to the skin around the abdominal region as shown in Fig. 5A. Insulin was injected at different time points. We used multiple insulin boluse injections to decrease the blood glucose levels to hypoglycemia. The responses of the antennas were saved every 2.5 min for a period of 2 h. Reference glucose levels were measured at different times using a commercial glucometer. The sensors’ S11 variations showed clear and linear relationships with the glucose variations (~ 10–100 mg/dl equivalent to 0.555- 5.55 mmol/l) demonstrating the potential of the proposed sensors to cover the hypoglycemic range, as shown in Fig. 5B,C, with a correlation of r > 0.9 for both sensors. In all three tested animals, CEG analysis revealed an improvement in the prediction when both sensors are used simultaneously as shown in Fig. 6 with a MARD of only 2.8% for the multi-sensing system compared with 3.9% MARD for the EM hand sensor and 5.8% for the EM leg sensor when used separately. Figure 6B shows the Clarke error grid analysis for the hand antenna alone (Left), leg antenna alone (middle) and both antennas. 100% of the predictions fall in the clinically acceptable Zones (Zones A and B) for the three scenarios, with the scenario where both sensors are used shows a superior performance compared with the standalone EM-sensors.

### In vivo monitoring on healthy individuals and patients with diabetes using the multi-sensing system

To evaluate the performance of the proposed multi-location, multi-sensor system, a clinical trial was carried out on 10 healthy subjects and 18 patients with diabetes. Signals from the hand and EM leg sensors along with the integrated environmental sensors were monitored, while varying the blood glucose levels, during a two-hour OGTT for everyone. One of the study goals is to check the effect of the ambient temperature, humidity, skin temperature, galvanic skin response and, movement on the RF-signal and to compensate for these perturbations.

**Figure 7:**
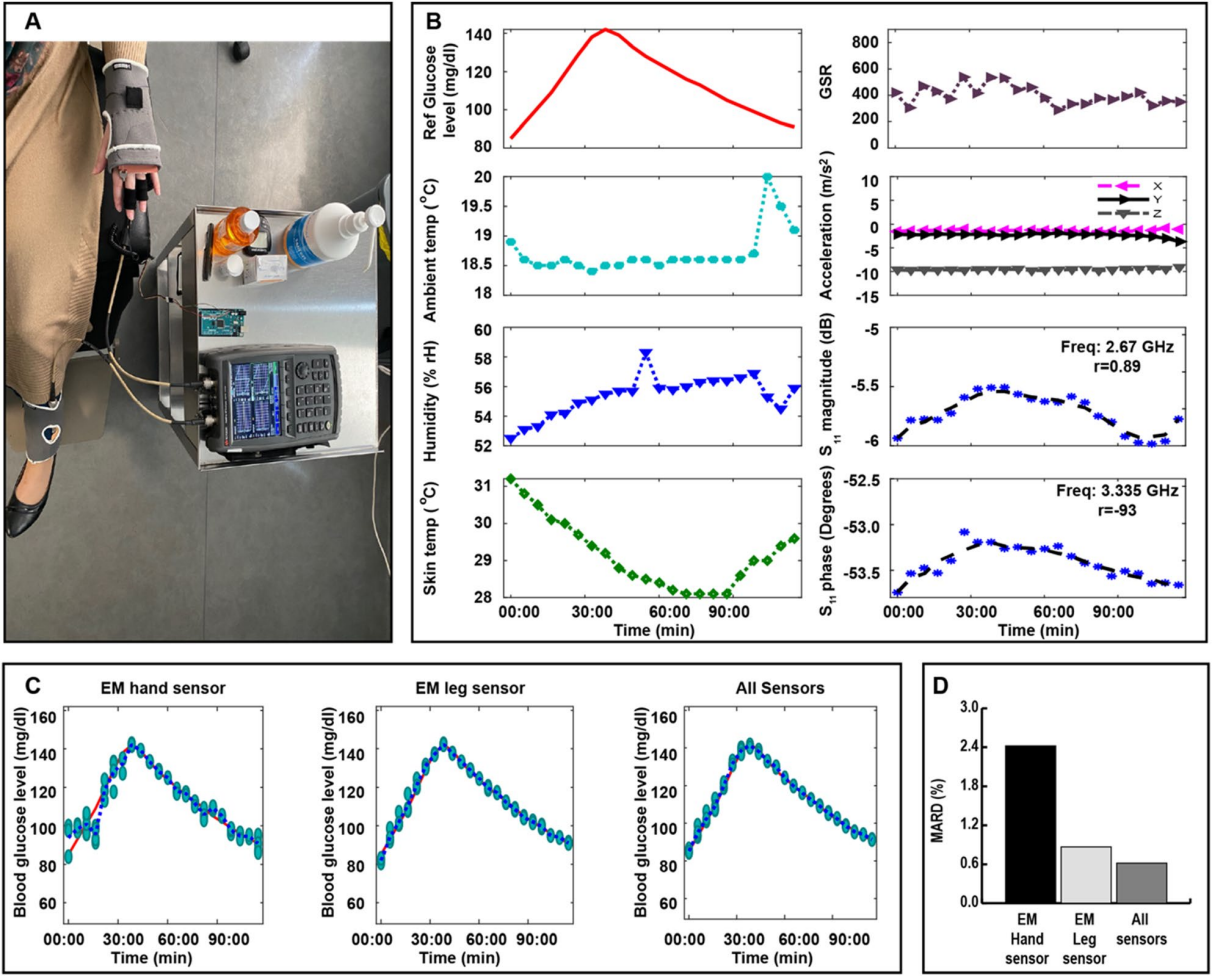
On body real-time monitoring of glucose levels during Oral glucose tolerance tests on diabetic and non-diabetic volunteers. (**A**) Photographs of a subject wearing a ‘smart glove and a ‘smart sock during an OGTT. (**B**) The collected on-body readings during one OGTT. (**C**) left: Glucose prediction using the GP model when relying only on the EM hand sensor data. Middle: Glucose prediction using the GP model when relying only on the EM leg sensor data. Right: Multi-sensing system blood glucose estimation. (**D**) Comparison between the standalone and combined system in terms of mean percentage error of the glucose levels prediction.

The responses, towards the variation of glucose levels, collected from on-body readings during one OGTT using the multi-sensing system are detailed in Fig. 7. Normalized data collected from the environmental/physiological sensors: skin temperature, humidity, ambient temperature, GSR and motion in terms of X, Y and Z are measured as shown in Fig. 7A. Additional discussion is presented in supplementary note 11.

These signals along with the S parameters collected from the EM leg and hand sensors, shown in Fig. 7B, are used to predict the glucose levels. Using the wrapper feature-selection technique, we can identify the important features for everyone by analyzing their data. A Gaussian process regression model^38,39^ is adopted and each OGTT is processed separately. The comparison between the standalone and combined system in terms of mean percentage error of the glucose levels prediction shows that the multi-sensing system provided lower prediction error compared with the standalone system as displayed in Fig. 7C,D.

**Figure 8:**
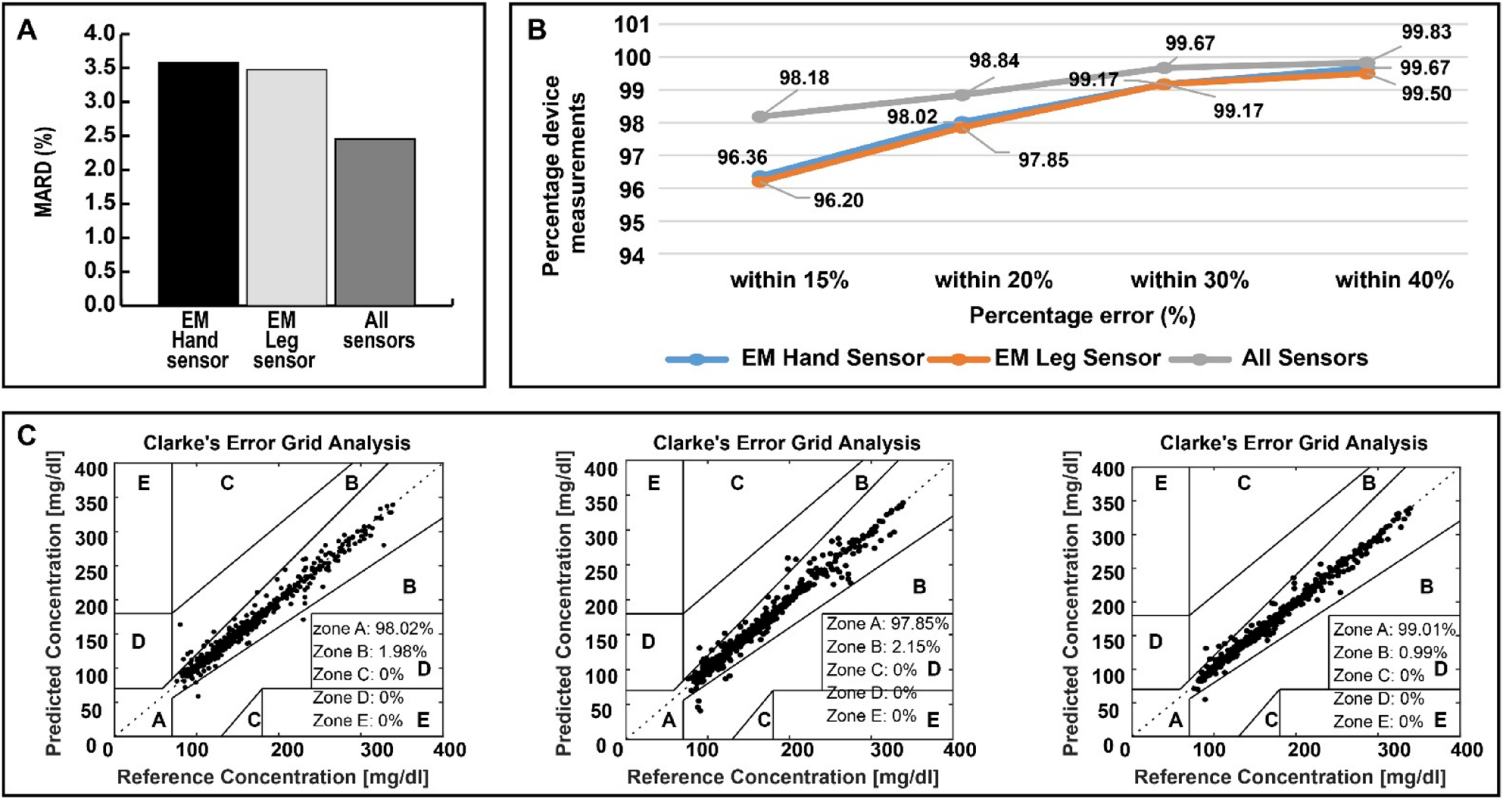
Comparison between the standalone and combined multi-location system. (**A**) MARD for the 28 experiments. (**B**) Percentage of device measurements with 15%, 20%, 30% and 40% error. (**C**) CEG for all the OGTT conducted by the 28 volunteers for the standalone systems and the combined system.

In the same context, Fig. 8 shows a comparison between prediction errors for the EM hand sensor used alone, EM leg sensor used alone and the combined system. CEG for all the OGTT conducted by the 28 individuals for the standalone systems and the combined system. Additional results are provided in Supplementary Figs. S7–S10. All the estimated values are in the clinically acceptable zones A and B with the majority in zone A.

The EM hand sensor resulted in 98.02% of the predictions in zone A and the remaining 1.98% of the prediction points in zone B. For the EM leg sensor, used alone, 97.85% of the predictions fall in zone A whereas the combined system, which includes both EM sensors and the physiological and environmental sensors, resulted in 99.01% of the predictions in zone A. For the combined multi-sensing system: 98.18% of estimated values are within the 15% MARD error, 98.84% are within 20% MARD error, 99.67% are within 30% MARD error and 99.83% are within 40% MARD error as detailed in Fig. 8B.

The multi-sensing system displays a superior performance compared with the standalone EM-sensors. If we compare the MARD values obtained by these three setups, we conclude that the combined multi-location multi-sensing system provided the lowest MARD as shown in Fig. 8A. The multi-location, multi-sensing system reduces the impact of interfering factors in real-life condition. However, we also note that each sensor can be used alone, providing predictions in the clinically acceptable zones, as shown in the CEG in Fig. 8C.

## Discussion

In this study, we propose a non-invasive continuous multi-modality system coupled with a machine learning-aided signal processing interface that can monitor glucose concentrations over the hypo-to hyperglycemic range during clinical trials. Adding environmental and physiological sensors enhances the accuracy of the EM ultra-flexible sensors. Additionally, the topology of the proposed EM sensors mimicking the blood vessels network, the quasi-antenna-array form factor, the operational frequencies, and the on-body matching characteristics permitted us to track glucose variations with high fidelity during clinical trials, which had been undetectable using conventional non-invasive techniques. In terms of MARD and CEG, the proposed system in this work provides low error and good prediction accuracy. While advancements were recently made in noninvasive glucose monitoring using EM technology, none of these previous approaches underwent clinical trials. The proposed system in this work offers for the first time an environmentally and physiologically aware device, with the topology tailored towards the veins and arteries exhibiting a powerful capability that achieves high-resolution continuous glucose monitoring directly and wirelessly from the blood with picomolar sensitivity. Our unique design approach considers the ease and comfort of the patient as a top priority as demonstrated in the conducted clinical trials. In-vitro experiments not only evaluated and demonstrated the sensitivity and selectivity of the proposed EM-sensors towards the glucose variations but also tested their continuous endurance for an extended experimental duration up to 8 h.

Experiments on anaesthetized animal models confirmed the system’s ability to continuously monitor in vivo changes in glucose and highlighted the ability of the proposed system to monitor hypo-to hyper glycemic levels in real time settings, a crucial feature for clinical implementation. Subsequent experiments in clinical trials on patients with diabetes and healthy controls demonstrated that our proposed system achieved sufficient sensitivity to detect the variations of concentrations of blood glucose, with an accuracy of 99.01%. Most importantly, the noninvasive measurements closely matched those obtained with standard glucose finger-prick glucometer. As a result, this work proves the validity, accuracy and sensitivity of wearable EM based sensor for continuous glucose monitoring. Beyond the exceptional performance of the sensors, the work presented here, prioritizes the user’s comfort without and preference without compromising any of the needed instantaneous tracking (see supplementary Note 12).

## Methods

### Electromagnetic simulation of the sensors and fabrication process

High frequency structure simulator, Ansys electronics desktop (AED), was used to simulate the electromagnetic behavior of the sensor loaded with human tissues as expected during usage. A 3D model of the sensor is designed and simulated using AED. The leg sensor comprises three layers: the top sensing layer composed of 4 elements with non-identical slots, matching the leg’s vasculature anatomy along the feeding network. The middle layer includes a flexible ultrathin substrate, and the bottom layer contains the ground plane. The details of size calculation of the proposed sensor and the feeding line structure are presented in supplementary note 3.

The antenna is designed to operate in the presence of a leg model parallel to the top layer side of the antenna with a 0.5 cm separation. For purposes of simulation, the human leg tissue comprises five layers: the skin layer, the fat layer, the blood layer, the muscle layer, and the bone layer. The dielectric properties and the thickness of the leg tissues are extracted from the IT’IS Database^40^ and set in AED. The proposed microstrip patch antenna quasi-array is fabricated on an ultrathin flexible substrate as a main sensing element in the multi-sensing non- invasive glucose monitoring system. To adapt the antenna structures to the curvature of the leg, these antennas are designed by relying on an ultrathin flexible PET substrate material of 100 um thickness with a dielectric constant of 3.1 where Copper is used as a conductive material. After optimization, the final dimensions of the patch antenna quasi- array are set to 17.5 × 5.5 cm^2^. The size is optimized for integration in a wearable sock.

### Experimental setup for glucose monitoring in serum

Real-time monitoring of glucose levels in fetal bovine serum (FBS) for the sensor is held at a stable room temperature. The readings are recorded using a portable Keysight Fieldfox vector network analyzer (VNA)^41^. FBS contained nutritional factors, growth factors, and small molecules including amino acids, sugars, lipids, and hormones^32^. Sequential additions and mixing of known concentrations of SIGMA D-glucose^42^ into the FBS contained in a foam container with a pipette led to linear shifts of S-parameters collected at several frequencies. Reference glucose levels were obtained using the Accu-Chek glucometer from Roche^33^. For each glucose addition, 10 recordings of the S-parameters magnitude and phase were saved using the VNA. A total of 40 measurements are taken for the leg sensor and covering the hypo- to hyperglycemic levels. Sensor selectivity studies involved the addition of 50 mg/dl (2.775 mmol/l) Metformin (MET), 50 mg/dl (2.775 mmol/l) Oleic Acid (OA), 50 mg/dl (2.775 mmol/l) Panadol (PAN), 50 mg/dl (2.775 mmol/l) Fructose (FRU) and 50 mg/dl (2.775 mmol/l) Glucose (GLU).

### Experimental setup on diabetic and non-diabetic rat models

Seven rats were included in this study. The rats were divided as follows: (i) Five female Long- Evans rats, three of them received intravenous streptozotocin (STZ) injection to render them diabetic, and (ii) two male Long-Evans rats. STZ-induced diabetic rat models were prepared by single intravenous STZ injection (55 mg kg^−1^) to NEW XEALANF white rats after 12 h of fasting. After 48 Hours, the rats with blood glucose levels higher than 300 mg/dl (16.65 mmol/l) were considered diabetic. This experiment was conducted in accordance with the United States Public Health Service's Policy on Humane Care and Use of Laboratory Animals guidelines and regulations applicable at the time and location of the experiment, including the ARRIVE guidelines^43^. The experimental protocol was approved by the Institutional Animal Care and Use Committee at the American University of Beirut. During these experiments, rats were first anesthetized using an intraperitoneal injection of a mixture of ketamine (100 mg/kg) and xylazine (10 mg/kg). After sedation, the leg sensor was fixed on the abdomen area. The architecture of the rat’s abdomen vessels has a certain similarity with the leg’s vessels’ architecture^44^. Signals from the leg sensor were recorded every 5 min and reference glucose levels were measured simultaneously every 5 min by taking a drop of blood from the tail of the animal and measuring the actual glucose level using a commercial invasive glucometer. To reduce the rat’s glucose levels, an intraperitoneal injection of insulin is given to reach hypoglycemic levels.

### Experimental setup on non-diabetic pigs

Three Dalland Pigs (2 females and 1 male) are anesthetized using a mixture of atropine (0.04 mg/kg), ketamine (22 mg/kg) and xylazine (2 mg/kg) injected intramuscularly (IM). After sedation, animals are intubated and received isoflurane (1–2%) vaporized in air and oxygen, through mechanical ventilation, to maintain anesthesia during the experiment. The EM hand and EM leg sensors were fixed on the abdominal region. At time 0, fasting glucose level is measured using a commercial invasive glucometer. The pigs are then given intraperitoneal (IP) injection of insulin (8 U) to induce hypoglycemia. The glucose variation is monitored every 5 min. Signals from the EM leg sensor were recorded every 2.5 min. We relied on cubic spline interpolation to populate the remaining reference glucose points over 2.5 min-intervals.

### Experimental setup of the multi-sensing system on healthy individuals and patients with diabetes

For this study, 28 individuals were recruited, 10 healthy individuals and 18 patients with diabetes (2 type-1 and 16 type-2, 50% male and 50% female). This clinical trial followed the guidelines of the Institutional Review Board (IRB) at the American University of Beirut and was approved by AUB IRB committee. Inclusive criteria: individuals were considered eligible for the study if they were between 18 and 70 years of age and able to provide informed consent. No restrictions on either race, sex, or ethnicity. Exclusive criteria included: Substance abuse, lactation, pregnancy, and being part of an interventional trial. Everyone participated in one oral glucose tolerance test. During this test, individuals are asked to come to the clinic after 8 h of fasting. The multi-sensing system is fixed on the corresponding locations (Hand and Leg) for a period of 2 h. The individuals are asked to drink a sugar solution (containing 75 g of sugar). Signals from the multi-sensing system are recorded every 5 min. While invasive glucose measurements were done using a commercial glucometer every 15 min. We relied on cubic spline interpolation to populate the remaining reference glucose points over 5 min-intervals. Patients with diabetes took their medications (metformin pills or insulin shots) during the experiment.

### Data collection and feature selection

Signals were collected from the EM sensors, ambient temperature and humidity sensors, skin temperature sensor, skin conductance response sensor and motion sensor simultaneously. From the EM sensors, S-parameters magnitude and phase are collected at multiple frequencies over a range extending from 0.5 to 4 GHz. The recorded signals from all the sensors represent at total of ~ 80 features. First, the 10 S-parameters recordings obtained for each reference measurement are averaged. After that, all the features are normalized to a value between 0 and 1. These features are then integrated in a multivariate regression model to predict the absolute glucose values. Given the size of the dataset for each experiment, wrapper feature selection technique is utilized to identify the feature set exhibiting the highest sensitivity towards the glucose variations and hence avoid over fitting.

### Regression modeling techniques

Gaussian process regression (GPR)^38,39^ is utilized for glucose estimation using the selected features. The Gaussian process is a non-parametric kernel-based approach. It shows good performance when applied to small datasets. The sample points are related to each other using a covariance function k(x, xq). Different covariance functions were tested and the one providing the lowest mean percentage error was selected^38,39^. To train the model we relied on 2/3 of the dataset selected randomly. The remaining 1/3 is used for testing. This procedure is repeated 10 times to compensate for the small size of the datasets.

## Supplementary Information

Supplementary Information.

## Data Availability

All data generated or analyzed during this study are included in this published article and its supplementary information files. Additional data related to this paper may be requested from the authors.
