# Supplementary material for: Wearable flexible body matched electromagnetic sensors for personalized non-invasive glucose monitoring
Source: Sci Rep. 2022 Sep 1;12:14885. doi: 10.1038/s41598-022-19251-z (PMC9436982; doi:10.1038/s41598-022-19251-z)
Supplement: Supplementary file 1 — Supplementary Information. [file 41598_2022_19251_MOESM1_ESM.docx]

Supplementary Materials for

Wearable Flexible Body Matched Electromagnetic Sensors for Personalized Non-Invasive Glucose Monitoring

Jessica Hanna, Youssef Tawk, Sami Azar, Ali H. Ramadan, Batoul Dia, Elias Shamieh, Sumaya Zoghbi, Rouwaida Kanj*, Joseph Costantine*, Assaad A. Eid*.

**Contents**

[**Supplementary Note 1. Importance of noninvasive continuous glucose monitoring** 3](#_Toc108255852)

[**Supplementary Note 2. Depth of penetration (DOP)** 3](#_Toc108255853)

[**Supplementary Note 3. EM- sensor design details** 4](#_Toc108255854)

[**Supplementary Note 4. EM- sensor parameters** 4](#_Toc108255855)

[**Supplementary Note 5. Response to common interferants** 4](#_Toc108255856)

[**Supplementary Note 6. Response to movements** 5](#_Toc108255857)

[**Supplementary Note 7. SAR** 5](#_Toc108255858)

[**Supplementary Note 8. Data collection and preprocessing** 5](#_Toc108255859)

[**Data collection (Pre-processing):** 6](#_Toc108255860)

[**Data Processing:** 6](#_Toc108255861)

[**Supplementary Note 9. Regression modeling** 6](#_Toc108255862)

[**Gaussian Process (GP)** 6](#_Toc108255863)

[**Derivation.** 6](#_Toc108255864)

[**Supplementary Note 10. In-vitro measurements of glucose with leg EM sensor.** 7](#_Toc108255865)

[**Supplementary Note 11. Multi sensing system** 8](#_Toc108255866)

[**Skin Temperature Sensor** 8](#_Toc108255867)

[**Skin conductance response (SCR) sensor** 8](#_Toc108255868)

[**Environmental temperature and humidity sensor** 8](#_Toc108255869)

[**Motion sensor** 8](#_Toc108255870)

[**Supplementary Note 12. Future work** 8](#_Toc108255871)

**Supplementary Notes**

# **Supplementary Note 1. Importance of noninvasive continuous glucose monitoring**

Some studies have shown that the tight glycemic control provided by the CGM systems reduces the levels of glycated haemoglobin (HbA1c). For instance, according to^1^, CGM reduces the number of hypoglycemic episodes for both adults and children. This study showed that the traditional invasive self-monitoring blood glucose (SMBG) techniques could miss 71% of hypoglycemic incidents when blood glucose levels are monitored four times per day^1^. This percentage is reduced to 58% missed episodes when the frequency of monitoring the glucose levels is increased to seven times per day^1^. HbA1c is also reduced by 0.5-1% for type 1 diabetes using CGM without increasing the number of hypoglycemic events^2^. Additionally, according to study^3^, continuous glycemic control in type 1 diabetes results in less microvascular and macrovascular incidences in the long term^3^. Most medthe ical professionals agree that diabetic patients treated with insulin should track their glucose variations at least four times a day^1^. However, several clinical studies assessing patients’ compliance to monitor glycaemia shows that the frequency of monitoring is subjective. Many, if not most, individuals with diabetes feel that the disadvantages of current commercial glucose monitoring technologies outweigh the advantages, which is why the compliance of testing is not ideal. A large portion of these individuals would prefer more freedom and flexibility from their glucose checking devices. One of the main barriers behind SMBG is the invasiveness of the available SMBG devices. Up to 63% of diabetic patients consider invasiveness the main reason behind skipping tests and would perform SMBG more frequently if a non-invasive technique exists^4^. This poor compliance with SMBG is not only related to the painful procedure but also includes the impact of the available SMBG techniques on the patient’s lifestyle and embarrassments caused by these devices being socially visible^3^.

Early efforts to this end have resulted in minimally invasive sensors, which rely on short-term microneedles inserted inside the skin. These sensors rely on amperometric enzyme electrodes that last only up to 14 days. They interpret the acquired data and translate them into dynamic plots providing information about the rate and the direction of glucose change during the day, month, and year. Despite its good accuracy, this approach is still considered uncomfortable and may cause irritation including local erythema, itching, inflammation and bleeding at the insertion site^5^. It can increase the patient’s resistance especially for elderly and kids, and most importantly, it still suffers from high socio-economic burdens^1,6^.

Hence, the ability to non-invasively monitor glucose variations with an easy to use and relatively low-cost technology would be crucial and game changing. Beyond that, there is a need to develop a wearable technology that is human-centered and positions the patient’s comfort and preference as a priority in the design process. Such a technology enhances the quality of life for millions around the world and allows clinicians to optimize diabetes therapeutic treatments. However, it remains extremely challenging to achieve CGM noninvasively.

Researchers studied several noninvasive glucose monitoring techniques including infrared spectroscopy^7^, ultrasound^8,9^, fluorescence^10^, optical coherence tomography^11^ and reverse iontophoresis^7,12^. Each of these solutions offer several advantages but all suffer from a number of technical challenges^13–17^. These technology-dependent limitations include lack of specificity, skin irritation, serious time lag when measuring glucose in the interstitial fluids, poor correlation between the measured glucose levels and the blood glucose levels when monitoring the glucose in tears or saliva and lack of stability due to interference from confounding factors^13–17^.

Dielectric spectroscopy is an approach where electromagnetic (EM) biosensors are studied for continuous, noninvasive glucose monitoring through skin and underlying tissues^18–^^23^. However, most of the proposed EM based systems neglect the effect induced by environmental and physiological factors. These perturbing factors include ambient temperature and humidity, skin temperature, skin conductance that is mainly affected by sweat, and motion which could affect the sensor-skin contact. Hence, taking into consideration all these different perturbing factors significantly enhances the accuracy and efficiency of EM based noninvasive glucose monitoring systems especially when tested on patients with diabetes.

# **Supplementary Note 2. Depth of penetration (DOP)**

A very important parameter to consider when choosing the operational frequency of the EM sensors is the depth of penetration (DOP). Any wave transmitted into a lossy material will be attenuated after a certain depth. The DOP, which is dependent on the MUT, decreases with the increase of the frequency. Specifically, the DOP is associated with the tissue-depending thermal effect. It is higher in the brain, fatty and bone tissues than in muscle tissues. At frequencies lower than 0.1 GHz, the wave can penetrate the skin, fat, and muscle. On the other hand, at frequencies higher than 10 GHz, the wave barely penetrates the skin, the fat, and the muscle layers. Additionally higher frequencies make the sensor more sensitive to motion and environmental factors. To optimize the performance of the proposed system, especially to measure glucose levels for different body types/compositions, the sensors are optimized to have a multi-band response in the ultra-high frequency (UHF), L, and lower S bands ranging between 500 MHz and 4 GHz. This frequency allows enough penetration for the waves to reach the targeted veins and arteries, for different layer thicknesses, while maintaining good sensitivity. The multi-band response provides a wide range of penetration depths. This is an important aspect that makes the sensor more suitable and easily adaptable for varying users' physiological needs such as age, gender, and body composition. Consequently, a different set of frequencies may be selected for different users.

# **Supplementary Note 3. EM- sensor design details**

The sensing layer is composed of four rectangular shaped conductive patches, as shown in the Figures 1 and 2. The size of these patches determines the operational frequency of the proposed microstrip patch antenna quasi-array. The width and length of these patches are calculated using the mathematical equations below^24^:

$W=\frac{c}{2\pi f}\sqrt{\frac{2}{\epsilon_{r}+1}}$ (1)

$\epsilon_{eff}=\frac{\epsilon_{r}+1}{2}+\frac{\epsilon_{r}-1}{2}\left( 1+12\frac{h}{W} \right)^{-1/2}$ (2)

$\Delta L=0.412\frac{h(\epsilon_{eff}+0.3)(\frac{W}{h}+0.264)}{{(\epsilon}_{eff}-0.258)(\frac{W}{h}+0.8)}$ (3)

$L_{eff}=L+2\Delta L$ (4)

$z=\frac{120\pi}{\sqrt{\epsilon_{eff}}\left[ \frac{w_{0}}{h}+1.393+0.667ln(\frac{W_{0}}{h}+1.444) \right]}$ (5)

where f is the antenna’s resonance frequency, W_0_ is microstrip line width, c is the EM waves velocity in vacuum, ɛ_r_ is the dielectric substrate’s relative permittivity, h is the antenna’s total height, L_eff_ is the patch’s effective length effective length of the patch.

After designing the patch, non-identical slots that mimic the blood vessels network of the leg are introduced to concentrate the EM waves onto the targeted veins and arteries. These integrated slots are optimized to operate with great matching at the desired frequencies of operation, while being loaded by the human leg model. The width of the slots are optimized based on the average diameters of the different blood vessels

A Microstrip line feeding network is utilized to power the antenna with impedance matching achieved by relying on Quarter-wave transformers (QWT) technique. The impedance of the QWT is calculated using the equation below^24^:

$$Z_{QWT}=\sqrt{Z_{i}. Z_{0}}$$

where Z_i_ and Z_0_ are impedance values of any microstrip lines within the QWT. The distances between the patches are 0.75 λ.

# **Supplementary Note 4. EM- sensor parameters**

The changes in the reflected waves, collected in terms of scattering parameters (S-parameters), are correlated to the glucose levels variations. The S-parameters are defined as input-output relationship between the terminals of the EM sensors. In particular, S11 represents the reflected power i.e. how much power is reflected from the antenna, and hence is known as the reflection coefficient/ return loss^24^.

# **Supplementary Note 5. Response to common interferants**

To evaluate the effect of some common interferants, including metformin, oleic acid, Panadol, and fructose, on the proposed sensor's response, glucose and these interferants were added to the FBS solution in concentrations much higher than their physiological ranges. We added successively 50 mg/dl of MET, OA, PAN, FRU and GLU to the same FBS solution. The S11 parameters showed minimal to no shift when the interferants were added as shown in Fig.3D. In contrast, a significant shift of S11 parameters is produced when the same amount of glucose is added to the solution, resulting in a correlation with the glucose levels of R > 0.9.

The difference in response is due to the difference in terms of dielectric properties between these interferents. In a lossy medium, containing lossy materials such as these interferents, the effective complex permittivity is composed of $\varepsilon_{r}^{'}$ which is the real permittivity that signifies the stored electric field energy and $\varepsilon_{r}^{''}$ which is the imaginary permittivity that accounts for the losses in the medium among which tanδ is the loss tangent of the medium. Hence, the complex permittivity highly affects the EM waves’ properties in terms of magnitude and phase.

# **Supplementary Note 6. Response to movements**

In a serum experiment, we prove that slight misalignment between the antenna’s slots and the vessels does not affect the sensitivity of the antennas. In this experiment, we introduced a slight misalignment (around 2 mm) between the slots and the vessel-like foam where the foam container was shifted to the right. We compared the results obtained in both experiments: the parallel position and the shifted position. The sensitivity of the sensor was preserved, and we were able to identify multiple frequencies at which the physical parameters of the proposed sensor (input features) stayed stable, demonstrating consistent and repetitive behavior even when a slight misalignment is introduced, as shown in the supplementary Fig.S3.

# **Supplementary Note 7. SAR**

One of the main concerns when it comes to designing a wearable EM sensor is the safety of the human body against the hazard effects of the proposed sensor. Hence, the major challenge when designing such sensors is to keep the EM exposure under the standard safety limits. For this purpose, the specific absorption rate (SAR), which is defined as the rate of absorbed energy within the unit mass of the human body when exposed to EM field, is calculated using the following equation^25^:

$SAR= \frac{\sigma\left| E \right|^{2}}{\rho} [W/kg]$

Where:

$\sigma$ : Tissue conductivity $(S/m)$

$E$ : Electric field density $(V/m)$

$\rho$ : Density of the biological tissue ($kg/m^{3})$

In this study, the 1-g average SAR distribution for the proposed sensor is illustrated in Fig.3E and Supplementary Figure S4. The leg sensor was tested when loaded with a homogeneous five-layer tissue model as well as for inhomogeneous and irregular human leg structure imbedded in ANSYS AED^26^.

The homogeneous human leg tissue comprises 5 layers: the skin layer, the fat layer, the blood layer, the muscle layer, and the bone layer, as shown in Supplementary Figure S.4A. The dielectric properties and the thickness of the leg tissues are extracted from the IT'IS^27^ and set in ANSYS AED^26^.

From the Supplementary Figure S4A, the obtained peak SAR value over the whole frequency range of interest, from 0.5 to 4 GHz, is 7.216e-5 W/Kg at 3.248 GHz averaged over 1 g of tissue when a homogeneous model was used (Supplementary Figure S4A). A peak SAR value of 2e-4 W/kg was obtained at 3.3 GHz when a nonhomogeneous structure is utilized (Supplementary Figure S4B). These values fulfill the US guidelines for general public exposure, which is defined by the Federal Communications Commission (FCC); The maximum average SAR value averaged over 1 g of tissue should be less than or equal to 1.6 W/kg.

It's important to mention that during the clinical trials, volunteers didn't report any discomfort and no visible effect of the EM waves on the skin is observed.

# **Supplementary Note 8. Data collection and preprocessing**

During experiments, the reflection coefficient S11 magnitude and phase of the antenna are collected at 21 equally spaced frequencies ranging between 0.3GHz and 4 GHz, every 5 minutes, resulting in a total of 42 features for each observation (corresponding to a reference glucose level). Ten recordings of the antenna’s response were averaged for each reference glucose level to reduce the random noise induced by the vector network analyzer improving the signal-to-noise ratio.

The collected reflection coefficients are normalized between 0 and 1 and then considered as input variables also known as features used to build the regression model.

During clinical trials, invasive glucometers were used every 15 min to collect the reference glucose levels. In order to synchronize between the S-parameters collected every 5 minutes and the reference glucose levels collected every 15 min, we relied on, cubic spine interpolation^28,29^.

Pseudo code explaining in detail the data collection and processing steps

Inputs:

X_pre:_  :Pre-processed S-parameters

$Y_{ref}$: Glucometer measurements

Outputs of the data pre-processing steps:

X_norm_: normalized feature vector

Y: reference glucose level evaluated at all sensor points

For each experiment,

## **Data collection (Pre-processing):**

- Collect from the EM sensor i^th^ observation data point every 5 minutes:

$X_{\mathrm{pre}}(i, :)=\{x_{\mathrm{pre}}^{j}(i, :)\}$ with a total of 42 features in $X_{\mathrm{pre}}$, where

$i\in[1, 23]$ represents the observation number,$j\in[1, 2]$ represents each of S11 magnitudes and phase respectively, and each $x_{\mathrm{pre}}^{j}$comprises 201 features obtained over the frequency range [0.5 -4] GHz,

- Collect using the standard invasive technique m^th^ reference glucose level data point every 15 minutes: $y_{ref}(m)$ where m$\in\left[ 1, 10 \right].$

## **Data Processing:**

- Use cubic spline interpolation for the $y_{ref}$ values to obtain a total of 23 reference glucose level points Y (corresponding to the 23 observation points of the EM sensor over the duration of the OGTT)

- $X_{s}=\{X_{S}^{K}\}$ is the set of ~ 42 feature vectors obtained by sampling $X_{\mathrm{pre}}^{j}$by selecting every 10^th^ variable at steps of 0.125 GHz. Hence the first set for k$\in[1, 21]$ $X_{S}^{K}=X_{pre}^{1}(: , \left( k-1 \right)\times10)$ are obtained from the S11 magnitude parameters. Likewise $X_{S}^{k}$ for k$\in\left[ 22, 42 \right]$ is obtained from the S11 phase data $X_{pre}^{2}$.

- Normalize each feature vector, $X_{S}^{K}$, as follows.

$X_{\mathrm{norm}}^{k} (i)=\frac{X_{S}^{k} (i)-min(X_{S}^{k} )}{max(X_{S}^{k} )-min(X_{S}^{k})}$

- $X_{\mathrm{norm}}=\{X_{norm}^{K}\}$

# **Supplementary Note 9. Regression modeling**

We relied on patient-specific glucose prediction models. The glucose regulatory process for every patient is different. An individual model was created for each patient. The best feature set is then identified using the wrapper feature selection technique. The optimal feature subset is selected based on the regression model output. In this work, we relied on the forward feature selection (FFS) wrapper method where we start with zero features in the subset, and for each iteration, we add the feature providing the lowest model output mean error. For this purpose, 10-fold cross validation is utilized to minimize the feature subset selection bias. For this purpose, 10-fold cross-validation is utilized to minimize the feature subset selection bias. Once the optimal feature set is identified, the dataset is divided into a training set (2/3) and a testing set (1/3). A model was created using Gaussian process regression by relying on the training data, and the performance of this model was evaluated using the testing data. To cover all the observation points, we repeated this process 10 times by randomly splitting the data each time into training and testing sets. To evaluate the performance of the estimated glucose levels, evaluation criteria frequently used in the diabetes research community are implemented. These assessment criteria include the mean absolute relative difference (MARD)^30^ and the Clarke error grid (CEG)^31^.

## **Gaussian Process (GP)**

GP is a probabilistic modeling technique that utilizes a “distribution across an infinite function space to determine functions that are concordant with the underlying training data set” ^32^. It is a non-parametric kernel-based probabilistic technique. GP relies on covariance functions to find the similarities between the input feature sets and produce the output (Y).

**Derivation.** The model parameters are estimated using the training set using the exact GP regression method. One approach for estimating the model parameters, which are the covariance function hyperparameters and the noise variance, is by maximizing the likelihood P(y|X). For the test set, the predicted glucose levels are estimated as follows^32^. If we assume additive input, the covariance of the glucose levels (y) is expressed as:

$$cov\left( y \right)=K\left( X,X \right)+\mu I$$

where $\mu$ is a noise term, K is the covariance function, and X represent the input features.

For any point in the test set, the joint distribution of the reference glucose level (y) and estimated glucose level ($y_{*}$) is written as follows.

$$\binom{y}{y_{*}} \sim N \left( 0, \left[ \begin{matrix} K\left( X,X \right)+ \mu I & X(X,X^{*}) \\ K(X^{*},X) & K(X^{*},X^{*}) \end{matrix} \right] \right)$$

This joint distribution is utilized to estimate the glucose levels for the GP regression using the training glucose levels:

$$p(y_{*}| X,y,x^{*}) \sim N(\bar{y}_{*}, cov(y_{*}))$$

Where the mean is calculated as follows:

$$\bar{y}_{*} \dot{=} \mathbb{E} \left[ y_{*} \right|X, y, x^{*}]=K\left( X^{*},X \right)\left[ K\left( X,X \right)+ \mu I \right]^{-1}y$$

And the covariance is calculated using the following equation.

$$cov\left( y_{*} \right)=K\left( X^{*},X^{*} \right)-K\left( X^{*},X \right)\left[ K\left( X,X \right)+ \mu I \right]^{-1}K(X,X^{*})$$

The mean can be also calculated by minimizing the least-squares problem, as described by Ebden^32^. Five GPR modules are built using the five kernels, and the kernel providing the lowest mean percentage error is selected for the estimation of the glucose levels.

Supplementary Figures S5 to S10 show the raw antenna responses and the prediction results for the different experimental setups from in-vivo on rats and pigs to clinical trials using the hand and leg sensor separately.

# **Supplementary Note 10. In-vitro measurements of glucose with leg EM sensor.**

To characterize the sensitivity of the proposed EM-sensor, we tested it first with fetal bovine serum^33^ (FBS) spiked with known concentrations of glucose. We introduced increased dose of glucose to the solution and used the proposed EM sensors to monitor glucose changes for approximately 8 hours. Over the course of the experiment, we started with a glucose level of 40 mg/dl that reached 500 mg/dl at the end of the experiment. The S11 raw responses of the antenna presented in Fig. 3A, B show that our proposed EM-sensor is capable of detecting picomolar changes in glucose levels. The raw data collected from our proposed noninvasive EM sensor closely follows the reference glucose levels obtained by a commercially available finger-prick glucometer^34^.

As a result, the proposed sensors can achieve a correlation of 0.96 and 0.99 between the reference glucose levels, S11 magnitude and S11 phase respectively as shown in Fig.3 A and B. Beyond regular glucose changes, this experiment highlights the ability of the proposed EM sensor to detect glucose levels that vary from hypoglycemic to hyperglycemic ranges. We were able to identify multiple frequencies at which the physical parameters of the proposed sensor exhibited a heightened sensitivity towards the glucose variations. This has essential implications for patients, who are naturally diverse, and have different underlying tissue compositions and hence respond differently to the EM-waves application. Thus, it is important to provide personalized monitoring and find the glucose-sensitive frequency that suites each patient. Through this approach, the patient has been put at the center of the design.

In addition, we developed a custom signal-processing module to process and predict the glucose levels based on the data collected from the different sensors (See Data collection and feature selection in the methods). Fig. 3C shows the prediction results for the leg sensor. Fig. 3C left shows the mean percentage error in function of the number of features used by the model for the different kernel functions using the wrapper method as a feature selection technique (See Data collection and feature selection in the methods). It’s clear that the mean percentage error, or cross validation error, decreases when more features are added to the model until it reaches a minimum value and then it starts to increase again. The wrapper technique resulted in a mean percentage error, which dropped, from 9 % to around 4 % for the best number of features and kernel function. Fig. 3C right presents estimated glucose levels versus the reference glucose levels using Gaussian process regression models for the leg sensor. The squared exponential kernel provided the lowest mean percentage error using 15 features, achieving a mean absolute relative difference (MARD)^30^ of only 8.25%.

# **Supplementary Note 11. Multi sensing system**

Previous clinical trial's results showed good correlation between the dielectric signal and the actual blood glucose levels with very promising accuracy^35^. However, in daily-life situations, the dielectric spectroscopy could be affected by a variety of environmental and physiological factors. These perturbing factors include ambient temperature and humidity, skin temperature, skin conductance which is mainly affected by the sweat, and motion which could affect the sensor skin contact. As a result, any noninvasive glucose monitoring system based on EM technology must also take into consideration the different perturbing factors.

Hence, we introduce a strategy for fully noninvasive glucose monitoring system that allows the proposed EM technology to be integrated in a multi-sensing wearable format. The system is composed of two flexible EM-vasculature network-like sensors and multiple environmental and physiological sensors. The EM- based sensors are designed to monitor the glucose variations from different body locations simultaneously, integrated inside wearable apparels, and continuously monitor the glucose levels of deep vasculatures without the operational difficulties or instabilities encountered by other conventional approaches. To test and compensate for the possible influence of some environmental effects on the EM sensors readings, we created a sensing array comprising: Skin temperature sensor, sweat sensor, environmental temperature and humidity sensor and a motion sensor. All these sensors are embedded inside the designed glove and sock as shown in Figure 7A. Each sensor provides non-invasive and continuous monitoring of the different perturbing factors.

## **Skin Temperature Sensor**

Skin temperature can provide clinical information about many aspects of human physiology including various skin injuries and diseases^36^ which could affect the dielectric properties of the MUT. Additionally, skin temperature monitoring is needed to take into the account and reduce the temperature variation in the readings of the EM sensors through a built- in signal processor.

A Philips 21091A skin-surface temperature probe^37^ was used for skin temperature monitoring and fixed inside both the glove and the sock as. The accuracy of the probe is ± 0.1 °C for temperatures ranging between 25°C and 45 °C, and ±0.2 °C otherwise (0-60 °C). The collected data are sent via Bluetooth to a phone application.

## **Skin conductance response (SCR) sensor**

The SCR known also as galvanic skin response (GSR) measures the variation in the electrical conductivity of the skin which varies with its moisture level. These variations are mainly due to the skin sweat generated by the sweat glands and can be utilized to estimate the sweat rate. The GSR can be measured in terms of conductance, resistance and electro- physiological potential. The conductivity of the skin increases when the sweat rate increases^38^. The sensor measures the electrical conductivity between two electrodes and is considered a type of ohmmeter.

The GROVE GSR sensor^39^ is integrated inside the glove monitoring the skin conductivity using two Ag/AgCl electrodes fixed on the skin. A small voltage of 0.5 V is applied between the two electrodes, and by relying on ohm's law, the electrical conductivity of the skin is measured using the current flow between the two electrodes. The GSR sensor is wired to an Arduino to collect and save the data.

## **Environmental temperature and humidity sensor**

Since the environmental temperature and humidity may directly affect the electrical properties of the tissues under test specifically the dielectric constant and the loss tangent, compensation for these two factors is crucial in our application.

C. Jang et al.^19^ proved that the ambient temperature has a higher effect on the EM-sensor response than humidity.

To test and compensate for environmental temperature and humidity potential effects, a CC2650STK, Sensor Tag Development kit^40^ is added to the proposed system to monitor the ambient humidity and temperature of the test environment. The collected data are sent via Bluetooth to a phone application.

## **Motion sensor**

Motion could affect the EM-sensor to skin contact, hence to take into consideration these movements effect on the S-parameters readings, we added a FLORA Accelerometer sensor^41^ to the proposed system. The sensor is wired to an Arduino to collect and save the 3-axis accelerometer data.

# **Supplementary Note 12. Future work**

The next steps for the development of this wearable glucose monitoring system will focus on further miniaturizing the different components of the needed backend circuitry and providing efficient energy harvesting approach to sustainably power the proposed wearable system for long hours. With such modifications, the proposed system can provide an affordable fully wearable solution allowing noninvasive and continuous glucose monitoring throughout the day and night. This non-invasive, continuous monitoring of blood glucose level opens opportunities for personalized treatments and improve the patient’s compliance especially when the system aims to be embedded in their daily life wearables.

**Supplementary References**

1. Keenan, D. B., Mastrototaro, J. J., Voskanyan, G. & Steil, G. M. Delays in minimally invasive continuous glucose monitoring devices: a review of current technology. *J. Diabetes Sci. Technol.* **3**, 1207–1214 (2009).

2. Joubert, M. & Reznik, Y. Personal continuous glucose monitoring (CGM) in diabetes management: review of the literature and implementation for practical use. *Diabetes Res. Clin. Pract.* **96**, 294–305 (2012).

3. Overland, J. *et al.* Improving self-monitoring of blood glucose among adults with type 1 diabetes: results of the Mobile^TM^ study. *Diabetes Ther.* **5**, 557–565 (2014).

4. Wagner, J., Malchoff, C. & Abbott, G. Invasiveness as a barrier to self-monitoring of blood glucose in diabetes. *Diabetes Technol. \& Ther.* **7**, 612–619 (2005).

5. Food, U. S., Administration, D. & others. Summary of safety and effectiveness data (SSED). *Med. Device Database. http//www. fda. gov (accessed July3, 2019)* (2018).

6. Burge, M. R., Mitchell, S., Sawyer, A. & Schade, D. S. Continuous glucose monitoring: the future of diabetes management. *Diabetes Spectr.* **21**, 112–119 (2008).

7. Sieg, A., Guy, R. H. & Delgado-Charro, M. B. Noninvasive glucose monitoring by reverse iontophoresis in vivo: application of the internal standard concept. *Clin. Chem.* **50**, 1383–1390 (2004).

8. Lee, S., Nayak, V., Dodds, J., Pishko, M. & Smith, N. B. Glucose measurements with sensors and ultrasound. *Ultrasound Med. \& Biol.* **31**, 971–977 (2005).

9. Kost, J., Mitragotri, S., Gabbay, R. A., Pishko, M. & Langer, R. Transdermal monitoring of glucose and other analytes using ultrasound. *Nat. Med.* **6**, 347–350 (2000).

10. Evans, N. D., Gnudi, L., Rolinski, O. J., Birch, D. J. S. & Pickup, J. C. Non-invasive glucose monitoring by NAD (P) H autofluorescence spectroscopy in fibroblasts and adipocytes: a model for skin glucose sensing. *Diabetes Technol. \& Ther.* **5**, 807–816 (2003).

11. Gabbay, R. A. & Sivarajah, S. Optical coherence tomography-based continuous noninvasive glucose monitoring in patients with diabetes. *Diabetes Technol. \& Ther.* **10**, 188–193 (2008).

12. Lipani, L. *et al.* Non-invasive, transdermal, path-selective and specific glucose monitoring via a graphene-based platform. *Nat. Nanotechnol.* **13**, 504–511 (2018).

13. Gabbay, R. A. New developments in home glucose monitoring: Minimizing the pain. in *Can J Diabetes* (2003).

14. Bruen, D., Delaney, C., Florea, L. & Diamond, D. Glucose sensing for diabetes monitoring: recent developments. *Sensors* **17**, 1866 (2017).

15. So, C.-F., Choi, K.-S., Wong, T. K. S. & Chung, J. W. Y. Recent advances in noninvasive glucose monitoring. *Med. Devices (Auckland, NZ)* **5**, 45 (2012).

16. Choi, H. Recent developments in minimally and truly non-invasive blood glucose monitoring techniques. (2017).

17. Vashist, S. K. Non-invasive glucose monitoring technology in diabetes management: A review. *Anal. Chim. Acta* **750**, 16–27 (2012).

18. Jang, C., Park, J.-K., Lee, H.-J., Yun, G.-H. & Yook, J.-G. Non-Invasive Fluidic Glucose Detection Based on Dual Microwave Complementary Split Ring Resonators With a Switching Circuit for Environmental Effect Elimination. *IEEE Sens. J.* **20**, 8520–8527 (2020).

19. Jang, C., Park, J.-K., Lee, H.-J., Yun, G.-H. & Yook, J.-G. Temperature-corrected fluidic glucose sensor based on microwave resonator. *Sensors* **18**, 3850 (2018).

20. Govind, G. & Akhtar, M. J. Metamaterial-inspired microwave microfluidic sensor for glucose monitoring in aqueous solutions. *IEEE Sens. J.* **19**, 11900–11907 (2019).

21. Vélez, P. *et al.* Microwave microfluidic sensor based on a microstrip splitter/combiner configuration and split ring resonators (SRRs) for dielectric characterization of liquids. *IEEE Sens. J.* **17**, 6589–6598 (2017).

22. Saha, S. *et al.* A glucose sensing system based on transmission measurements at millimetre waves using micro strip patch antennas. *Sci. Rep.* **7**, 1–11 (2017).

23. Liu, W., Wang, M. & Shi, Y. A transmission-reflection method for complex permittivity measurement using a planar sensor. *IEEE Sens. J.* **18**, 4059–4065 (2018).

24. Balanis, C. A. *Antenna theory: analysis and design*. (John wiley \& sons, 2016).

25. IEEE. *IEEE Standard for Safety Levels With Respect to Human Exposure to Radio Frequency Electromagnetic Fields, 3 kHz to 300 GHz*. *IEEE Std C95.1-2005 (Revision of IEEE Std C95.1-1991)* vol. 2005 (2006).

26. Ansys. Ansys electronics desktop. (2021).

27. Yanamadala, J. *et al.* New VHP-Female v. 2.0 full-body computational phantom and its performance metrics using FEM simulator ANSYS HFSS. in *2015 37th Annual International Conference of the IEEE Engineering in Medicine and Biology Society (EMBC)* 3237–3241 (2015).

28. Leal, Y. *et al.* Real-time glucose estimation algorithm for continuous glucose monitoring using autoregressive models. *J. Diabetes Sci. Technol.* **4**, 391–403 (2010).

29. Bondia, J. *et al.* Using support vector machines to detect therapeutically incorrect measurements by the MiniMed CGMS®. *J. Diabetes Sci. Technol.* **2**, 622–629 (2008).

30. Pleus, S. *et al.* Performance evaluation of a continuous glucose monitoring system under conditions similar to daily life. (2013).

31. Clarke, W. L. The original Clarke error grid analysis (EGA). *Diabetes Technol. \& Ther.* **7**, 776–779 (2005).

32. Ebden, M. Gaussian Processes for Regression: A Quick Introduction. *GPtutorial. pdf* (2008).

33. Honn, K. V, Singley, J. A. & Chavin, W. Fetal bovine serum: a multivariate standard. *Proc. Soc. Exp. Biol. Med.* **149**, 344–347 (1975).

34. Accu-Chek Active blood glucose meter.

35. Hanna, J. *et al.* Noninvasive, wearable, and tunable electromagnetic multisensing system for continuous glucose monitoring, mimicking vasculature anatomy. *Sci. Adv.* **6**, eaba5320 (2020).

36. Webb, R. C. *et al.* Ultrathin conformal devices for precise and continuous thermal characterization of human skin. *Nat. Mater.* **12**, 938–944 (2013).

37. Philips. Skin Surface Temperature Probe disposable, sterilized, continuous monitoring. https://www.usa.philips.com/healthcare/product/HC21091A/skin-surface-temperature-probe-disposable--sterilized--continuous-monitoring-sensor.

38. Begtrup, G., Bertrand, J. A., Heikenfeld, J., Morgan, A. & Weinle, N. Sweat conductivity, volumetric sweat rate, and galvanic skin response devices and applications. (2019).

39. Seeed. Grove - GSR sensor. https://www.seeedstudio.com/Grove-GSR-sensor-p-1614.html.

40. Instruments, T. CC2650STK SimpleLink Bluetooth low energy/Multi-standard SensorTag.

41. Adafruit. FLORA Accelerometer/Compass Sensor - LSM303 - v1.0.

**Supplementary Figures:**


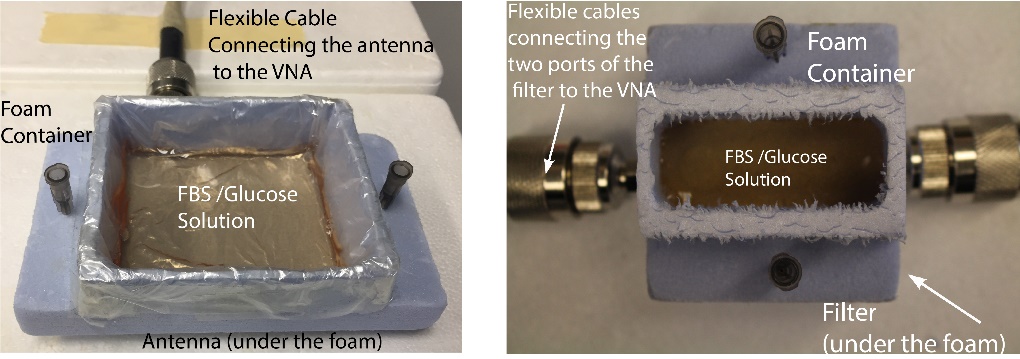


**Supplementary Figure S1. Response to glucose during serum-based experiments.** Schematic of the serum-based experiment.


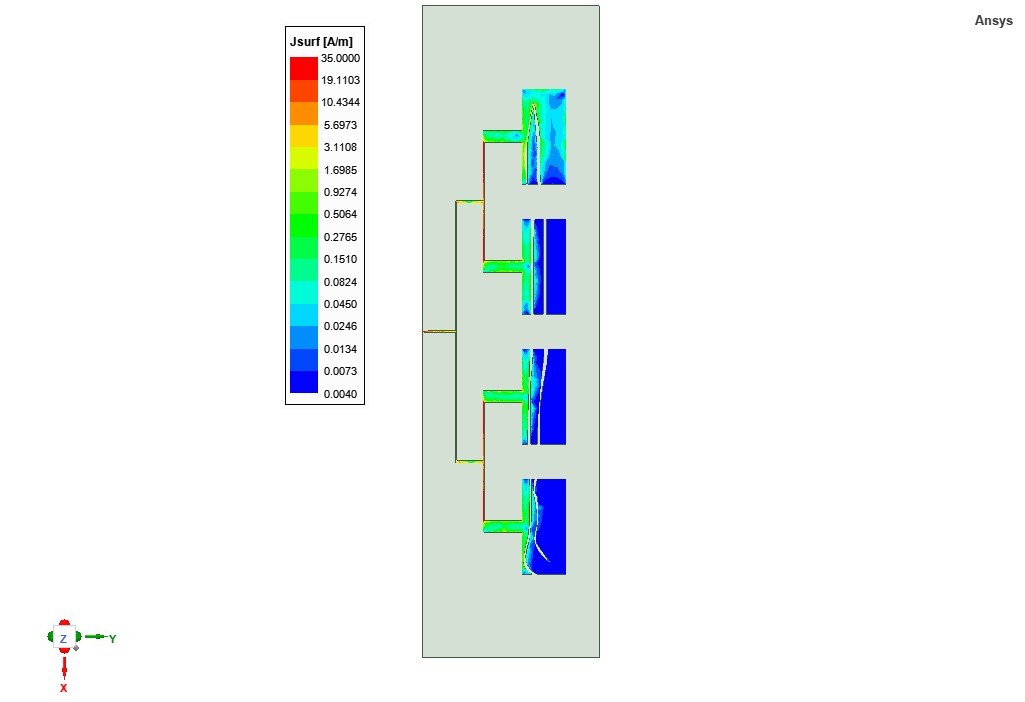

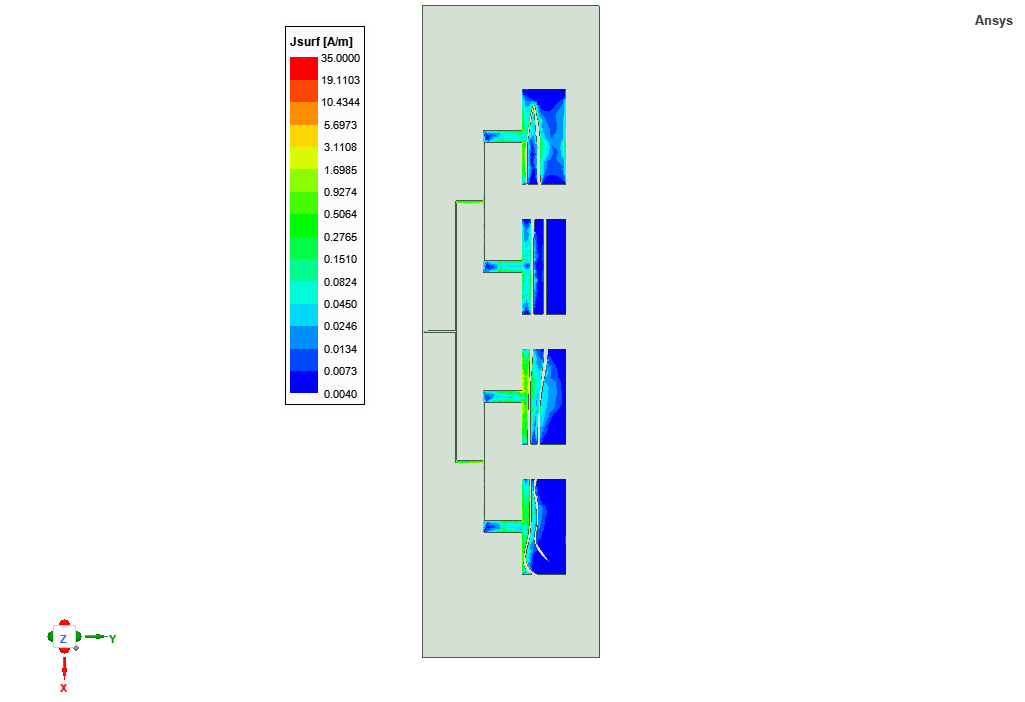


**Supplementary Figure S2. Surface Current Distribution of the flexible antenna at 0.1.875 GHz and 2.810GHz.**


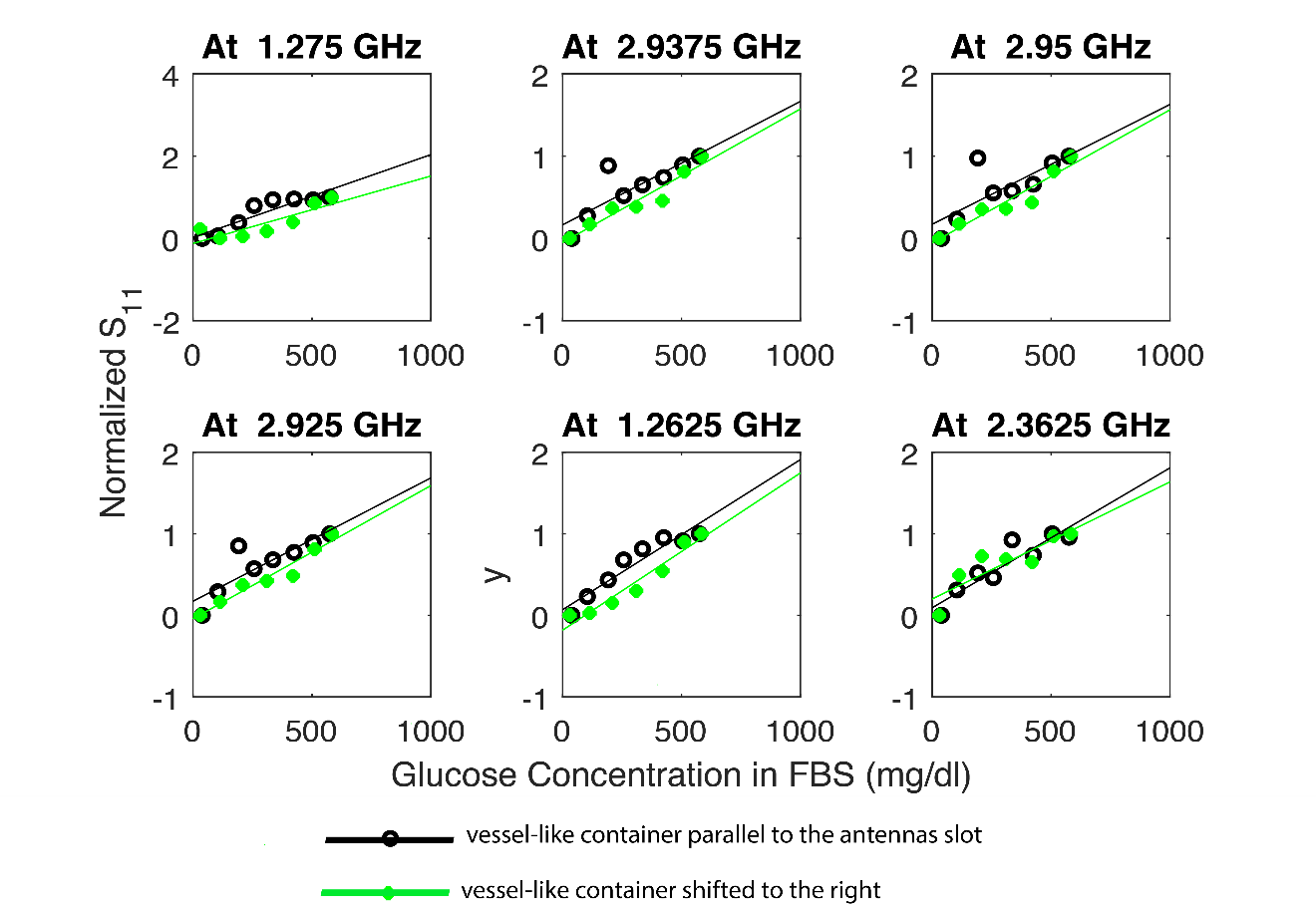


**Supplementary Figure S3. Small misalignment.** Multiple frequencies at which the physical parameters of the proposed sensor (input features) stayed stable, demonstrating consistent and repetitive behavior even when a small misalignment is introduced.


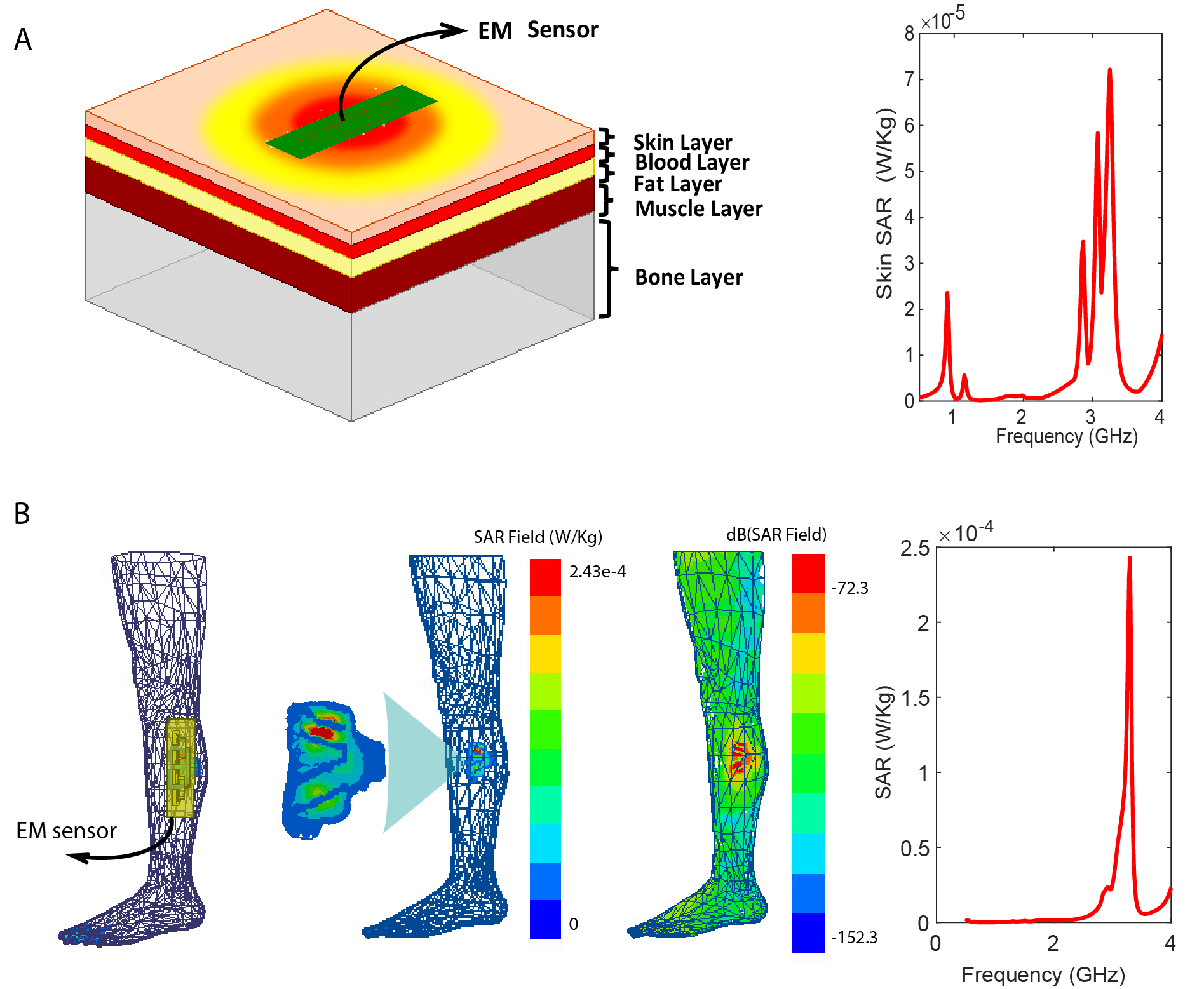


**Supplementary Figure S4. The 1-g average SAR distribution for the proposed leg sensor**. **A,** SAR simulated when the EM leg sensor is loaded with a homogeneous five-layer tissue model. The homogeneous human leg tissue comprises 5 layers: the skin layer, the fat layer, the blood layer, the muscle layer and the bone layer. Peak SAR value over the whole frequency range of interest, from 0.5 to 4 GHz, is 7.216e-5 W/Kg at 3.248 GHz averaged over 1 g of tissue. **B,** SAR simulated when the EM leg sensor is loaded with inhomogeneous and irregular human leg structure imbedded in ANSYS AED^26^. The peak SAR value over the whole frequency range of interest, from 0.5 to 4 GHz, is 7 2.43 e-4 W/kg was obtained at 3.3 GHz.

**
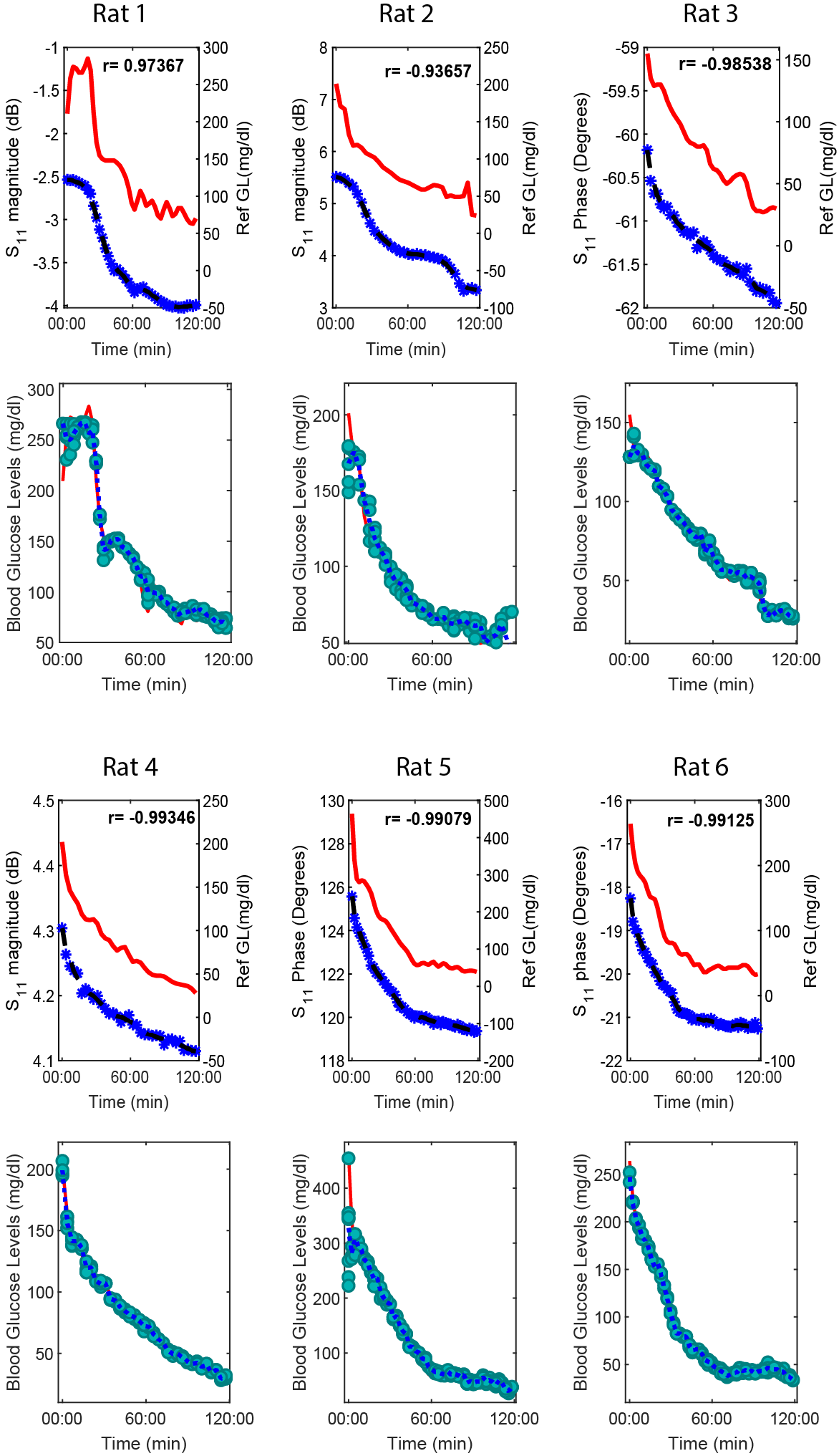
**

**Supplementary Figure S5. In-vivo experiments on rats using the leg sensor**. EM leg sensor’s response during 6 experiments done a diabetic and non-diabetic rat models. **Top,** The antenna’s raw S11 parameters in comparison with the reference glucose levels captured at different frequencies for each experiment. The red curve shows the reference glucose levels, the blue curve represent the antenna’s raw S11 response captured at a certain frequency. Theirs is a good correlation between the two values (r>0.9). **Bottom,** The predicted glucose level using the Gaussian Process regression technique for 10 randomly shuffled test/train (green dots show the estimations resulting from the 10 random repetitions, and the blue curve shows the mean estimation).

**
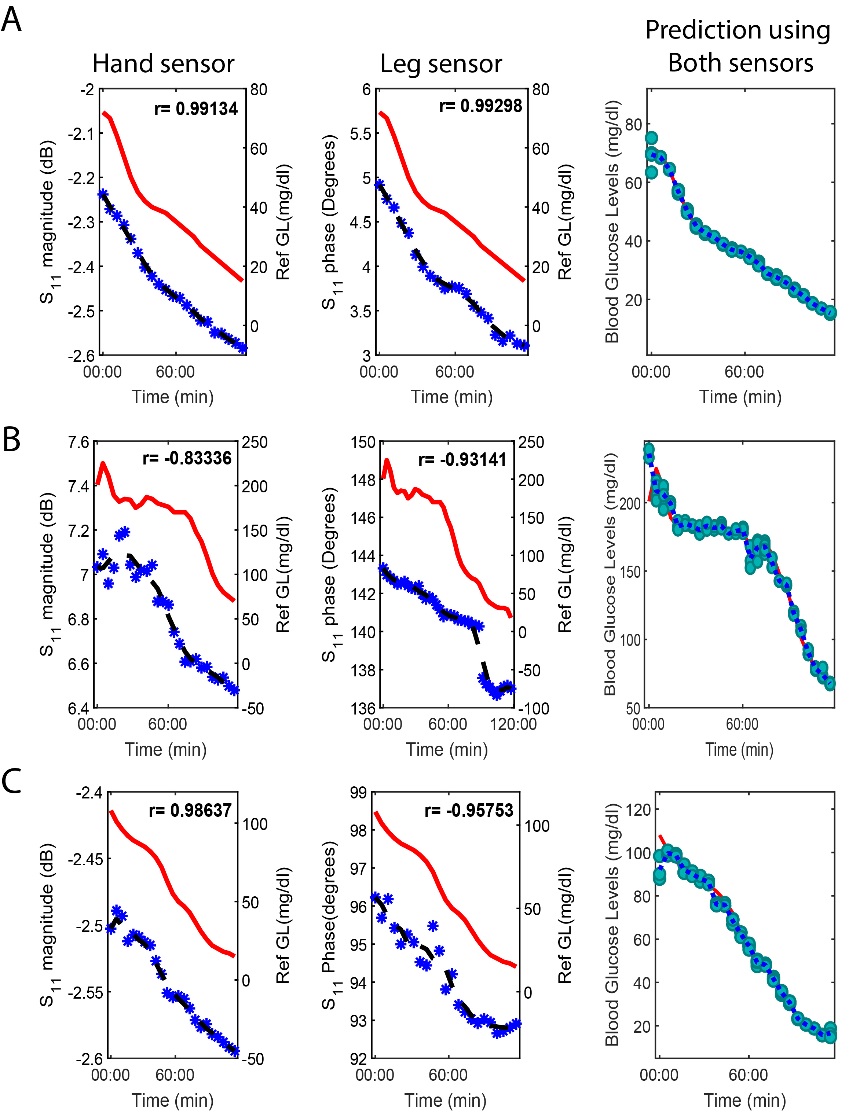
**

**Supplementary Figure S6. In-vivo experiments on pigs**. EM leg sensor’s response during 3 experiments done 3 non-diabetic pig models (A,B,C). **Left:** The EM hand sensor’s raw S11 parameters in comparison with the reference glucose levels captured at a selected frequency. The red curve shows the reference glucose levels; the blue curve represents the antenna’s raw S11 response captured at a certain frequency. **Middle:** The EM leg sensor’s raw S11 parameters in comparison with the reference glucose levels captured at a selected frequency. Theirs is a good correlation between the two values (r>0.9). **Right,** the predicted glucose level using the Gaussian Process regression technique for 10 randomly shuffled test/train (green dots show the estimations resulting from the 10 random repetitions, and the blue curve shows the mean estimation) when using the data collected from both sensors (leg and hand sensors).


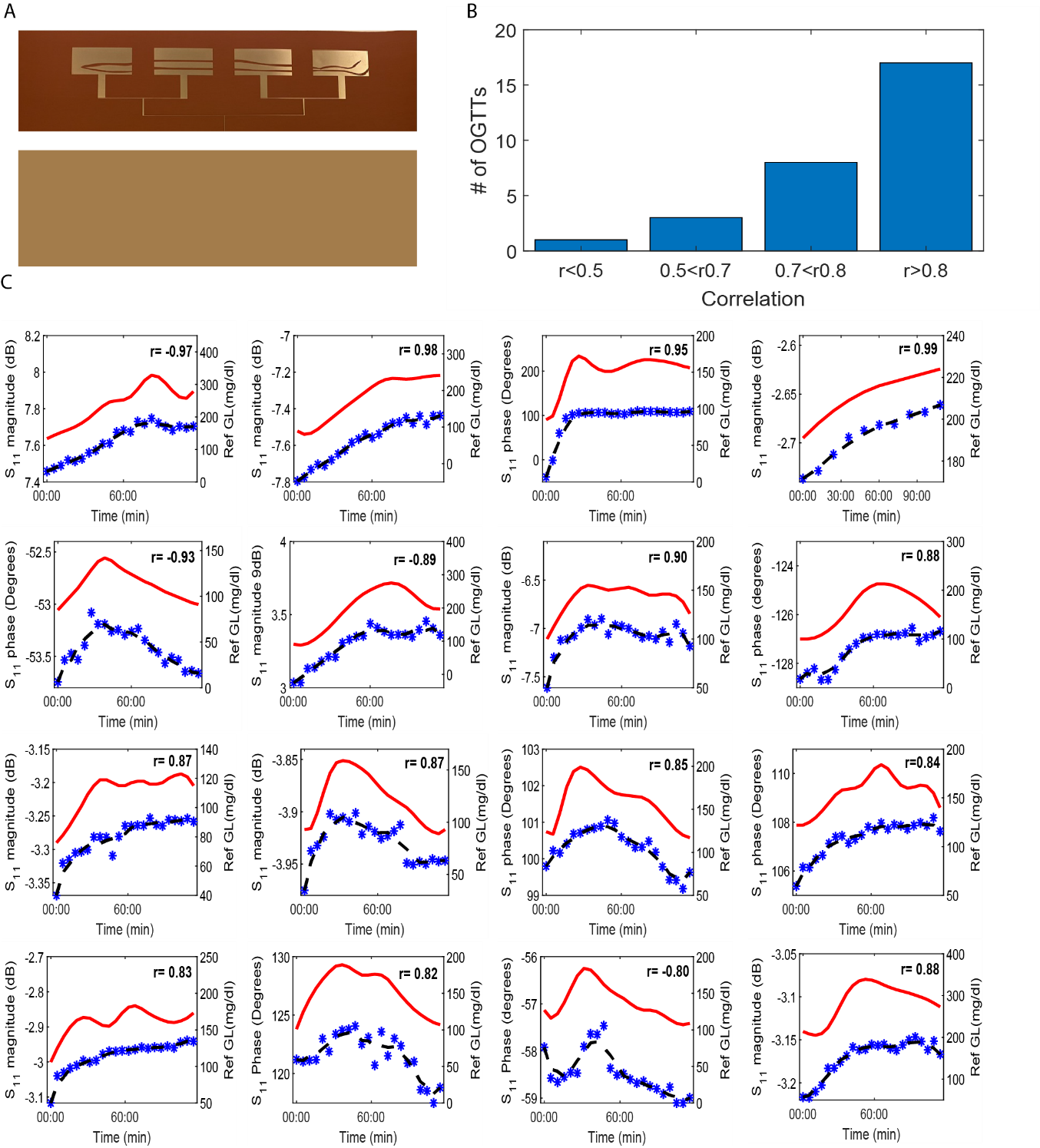


**Supplementary Figure S7. Human trials on healthy and diabetic volunteers using the EM leg sensor.** A, The fabricated flexible EM leg sensor. Top sensing layer (Top) and bottom layer (Bottom). B, The correlation between the antenna’s raw response (S-parameters; S magnitude and phase) and the reference blood glucose levels. High correlation were obtained for the majority of the OGTT experiments (r>0.8 for 17 out of 28 experiments). C, Antenna’s raw response in comparison with the reference glucose variation during representative 16 OGTT experiments chosen from the 8OGTTs. The blue curve represents the S-11 response versus time. The red curve shows the reference blood glucose profile versus time. High correlations is achieved between the two curves, indicating good tracking of the blood glucose variation during 2h span experiments. Additionally, no significant time lag is noticed between the antenna’s response and glucose peaks. Photo Credit: Jessica Hanna, American University of Beirut.


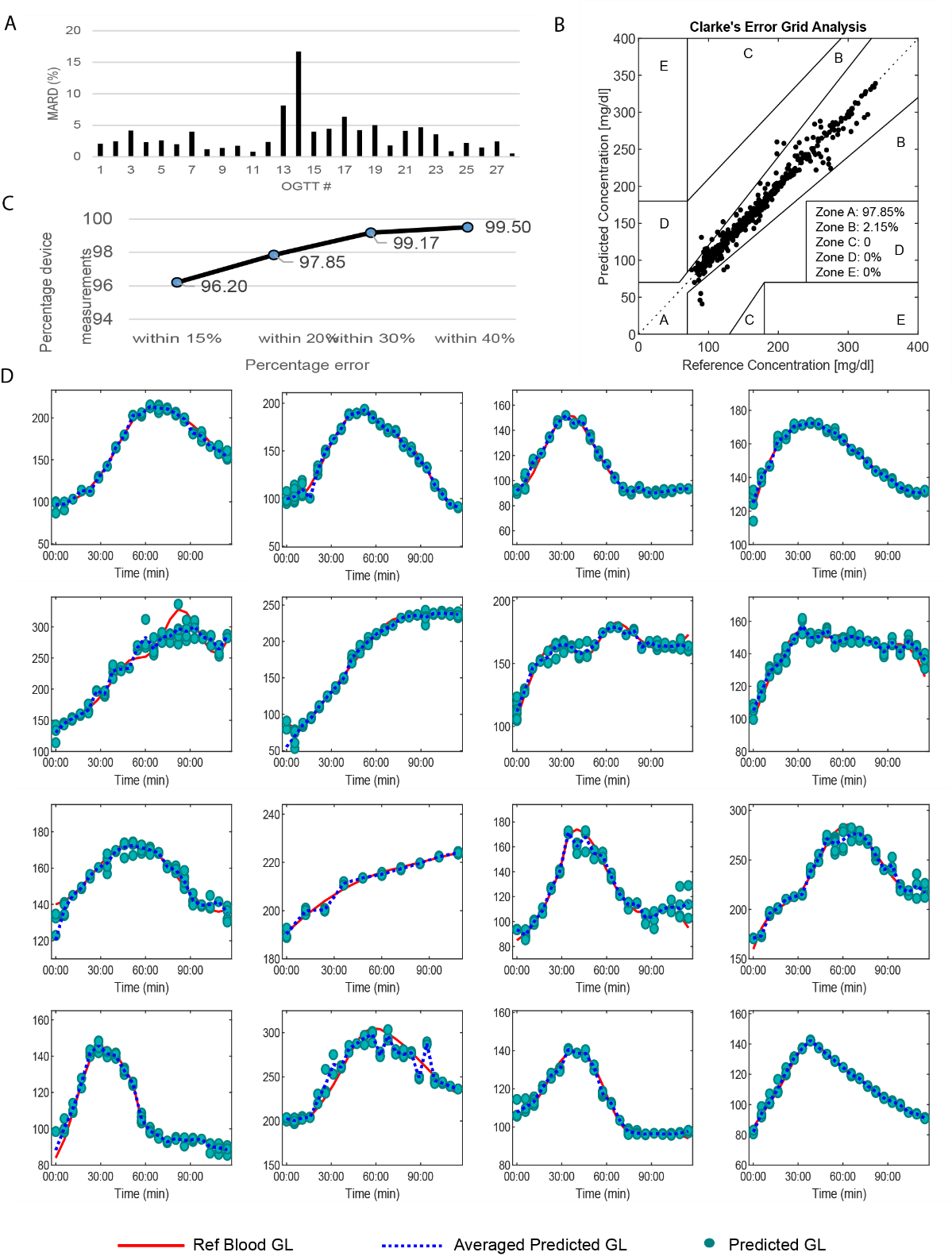


**Supplementary Figure S8. Predicted blood glucose levels during human trials using the EM leg sensor.** Gaussian Process regression technique was used to predict glucose levels using the data collected from the EM hand sensor in terms of S11 magnitude and phase**. A**, A Mean absolute relative difference of 3.47% was achieved for the 28 conducted OGTTs with a maximum value of 16.68%. **B**, The Clarke Error Grid analysis for the 28 experiments shows that all the averaged predicted points, of a total of 605 collected data point, falls in the clinically acceptable zones A and B: with 97.85% in zone A and the remaining 2.15% in zone B. **C**, 96.20 % of the device measurements fall within a 15% MARD, 97.85% within 20%, 99.17 within 30% and 99.50% within 40% MARD. **D**, Glucose prediction in comparison with the reference glucose variation during representative 16 OGTT experiments chosen from the 28 OGTTs. The green dots show the predictions resulting from the 10 random repetitions, and the blue curve shows the represents the averaged predicted glucose level and the red curve shows the reference blood glucose profile. Good agreement is achieved between the reference and the predicted values.


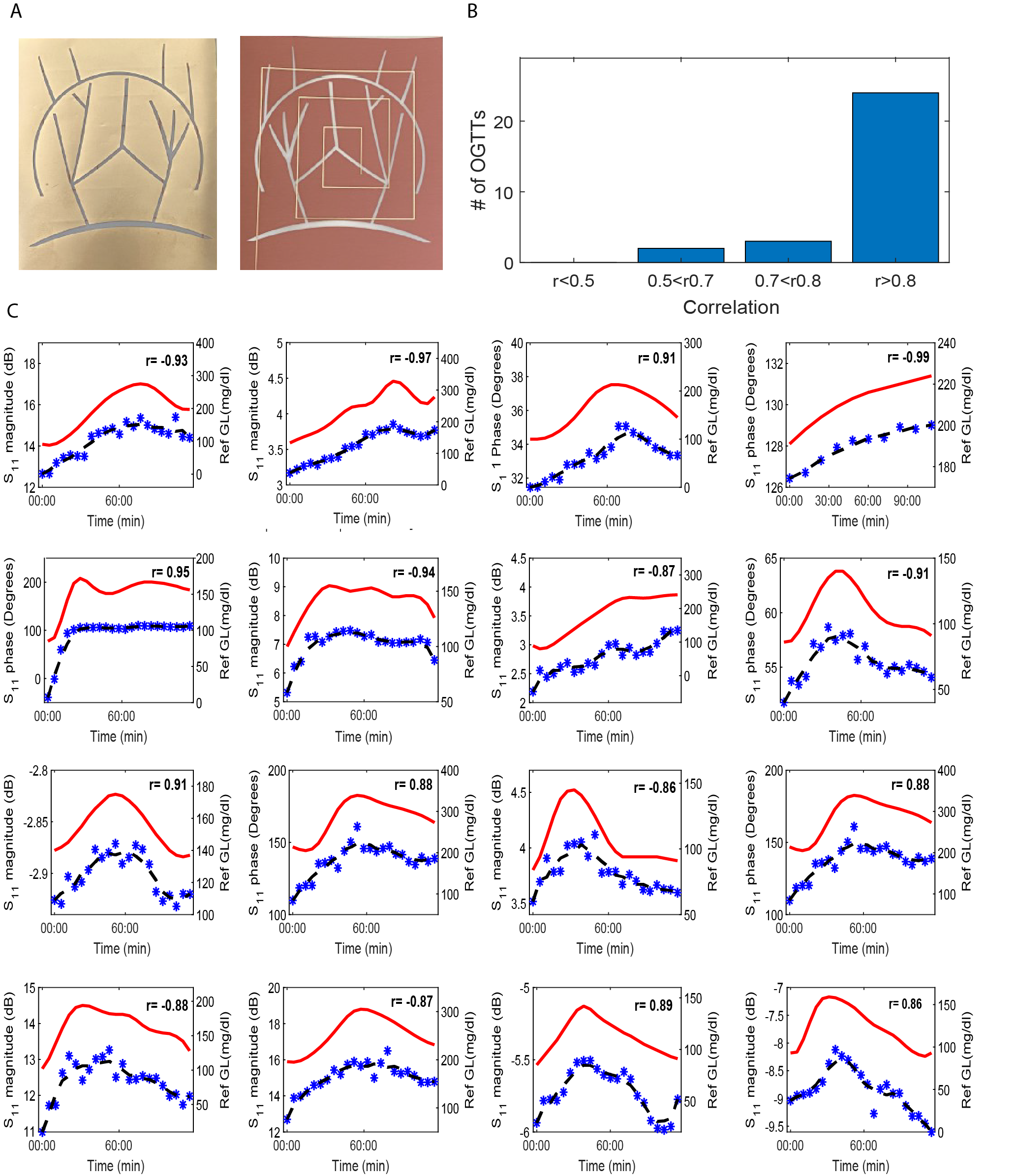


**Supplementary Figure S9. Human trials on healthy and diabetic volunteers using the EM hand sensor. A**, The fabricated flexible EM hand sensor. Left, Top sensing layer. Right, Bottom layer. B, The correlation between the antenna’s raw response (S-parameters; S magnitude and phase) and the reference blood glucose levels. High correlations were obtained for the majority of the OGTT experiments (r>0.8 for 24 out of 28 experiments). C, Antenna’s raw response in comparison with the reference glucose variation during representative 16 OGTT experiments chosen from the 28 OGTTs. The blue curve represents the S-11 response versus time. The red curve shows the reference blood glucose profile versus time. High correlations is achieved between the two curves, indicating good tracking of the blood glucose variation during 2h span experiments. Additionally, no significant time lag is noticed between the antenna’s response and glucose peaks. Photo Credit: Jessica Hanna, American University of Beirut.


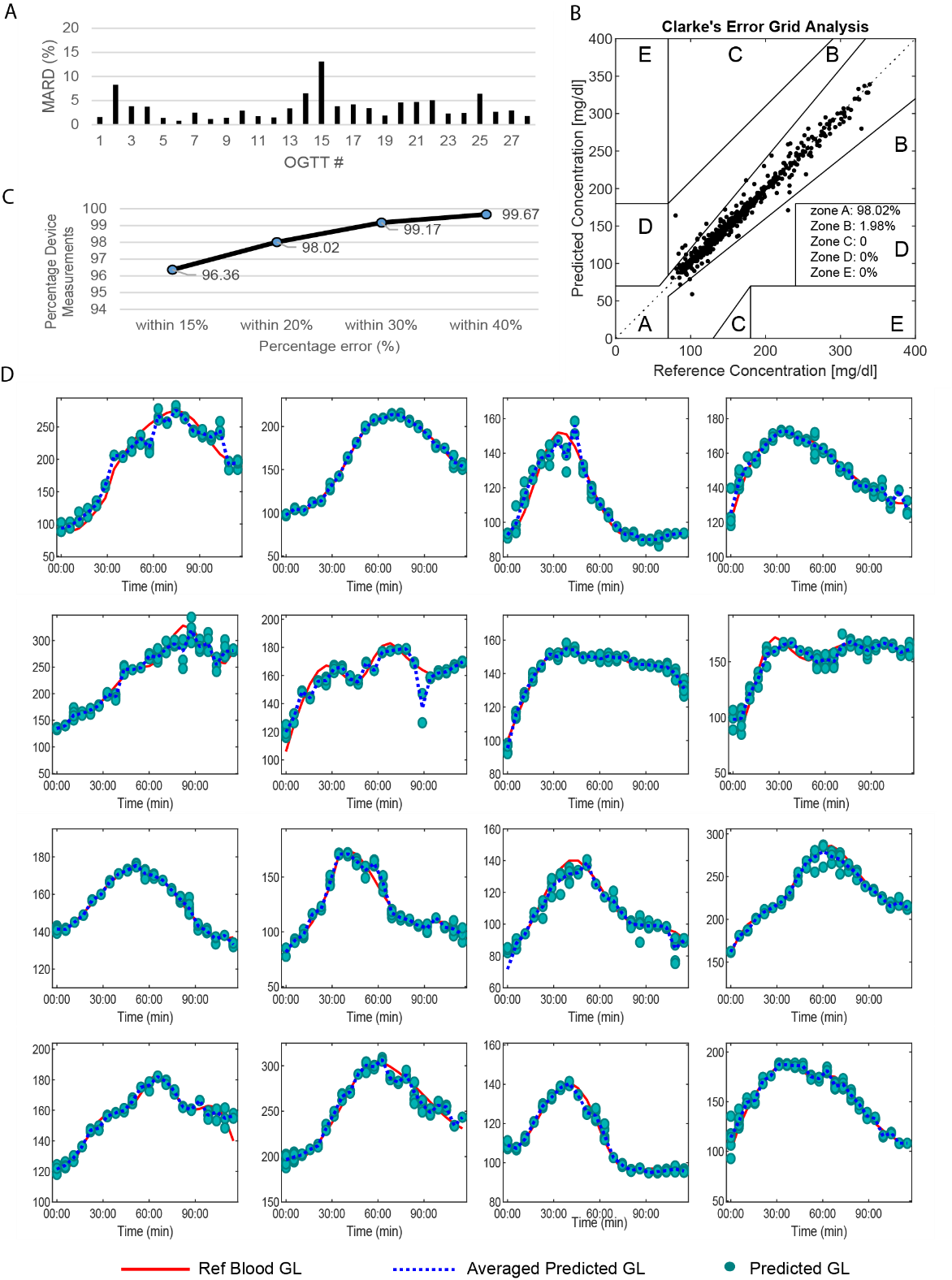


**Supplementary Figure S10. Predicted blood glucose levels during human trials using the EM hand sensor.** Gaussian Process regression technique was used to predict glucose levels using the data collected from the EM hand sensor in terms of S11 magnitude and phase**. A**, A Mean absolute relative difference of 3.58% was achieved for the 28 conducted OGTTs with a maximum value of 13.1%. **B**, The Clarke Error Grid analysis for the 28 experiments shows that all the averaged predicted points, of a total of 605 collected data point, falls in the clinically acceptable zones A and B: with 98.02% in zone A and the remaining 1.98% in zone B. **C**, 96.36 % of the device measurements fall within a 15% MARD, 98.02% within 20%, 99.17 within 30% and 99.67% within 40% MARD. **D**, Glucose prediction in comparison with the reference glucose variation during representative 16 OGTT experiments chosen from the 28 OGTTs. The green dots show the predictions resulting from the 10 random repetitions, and the blue curve shows the represents the averaged predicted glucose level and the red curve shows the reference blood glucose profile. Good agreement is achieved between the reference and the predicted values.
